# Pandemic *Vibrio cholerae* acquired competitive traits from an environmental *Vibrio* species

**DOI:** 10.26508/lsa.202201437

**Published:** 2022-11-29

**Authors:** Francis J Santoriello, Paul C Kirchberger, Yann Boucher, Stefan Pukatzki

**Affiliations:** 1 Department of Immunology and Microbiology, University of Colorado Anschutz Medical Campus, Aurora, CO, USA; 2 Department of Microbiology and Molecular Genetics, Oklahoma State University, Stillwater, OK, USA; 3 Saw Swee Hock School of Public Health and National University Hospital System, National University of Singapore, Singapore, Singapore; 4 Singapore Centre for Environmental Life Sciences Engineering, National University of Singapore, Singapore, Singapore; 5 Infectious Diseases Translational Research Program, Department of Microbiology and Immunology, Yong Loo Lin School of Medicine, National University of Singapore and National University Hospital System, Singapore, Singapore; 6 Department of Biology, The City College of New York, New York, NY, USA

## Abstract

*Vibrio cholerae* is a human pathogen that thrives in estuarine environments. Within the environment and human host, *V. cholerae* uses the type VI secretion system (T6SS) to inject toxic effectors into neighboring microbes and to establish its replicative niche. *V. cholerae* strains encode a wide variety of horizontally shared effectors, but pandemic isolates encode an identical set of distinct effectors. Effector set retention in pandemic strains despite mobility between disparate strains suggests that horizontal acquisition of these effectors was crucial for evolving pandemic *V. cholerae*. We attempted to locate the donor of the pandemic effectors to *V. cholerae*. To this end, we identified potential gene transfer events of the pandemic-associated T6SS clusters between a fish pathogen, *Vibrio anguillarum*, and *V. cholerae*. We supported the likelihood of interaction between these species by demonstrating that homologous effector-immunity pairs from *V. cholerae* and *V. anguillarum* can cross-neutralize one another. Thus, *V. anguillarum* constitutes an environmental reservoir of pandemic-associated *V. cholerae* T6SS effectors that may have initially facilitated competition between pre-pandemic *V. cholerae* and *V. anguillarum* for an environmental niche.

## Abstract (precis)

Pandemic-associated *Vibrio cholerae* interbacterial defense genes were likely acquired through competition with and subsequent gene transfer from the fish pathogen *Vibrio anguillarum*.

## Introduction

**Figure 1.**
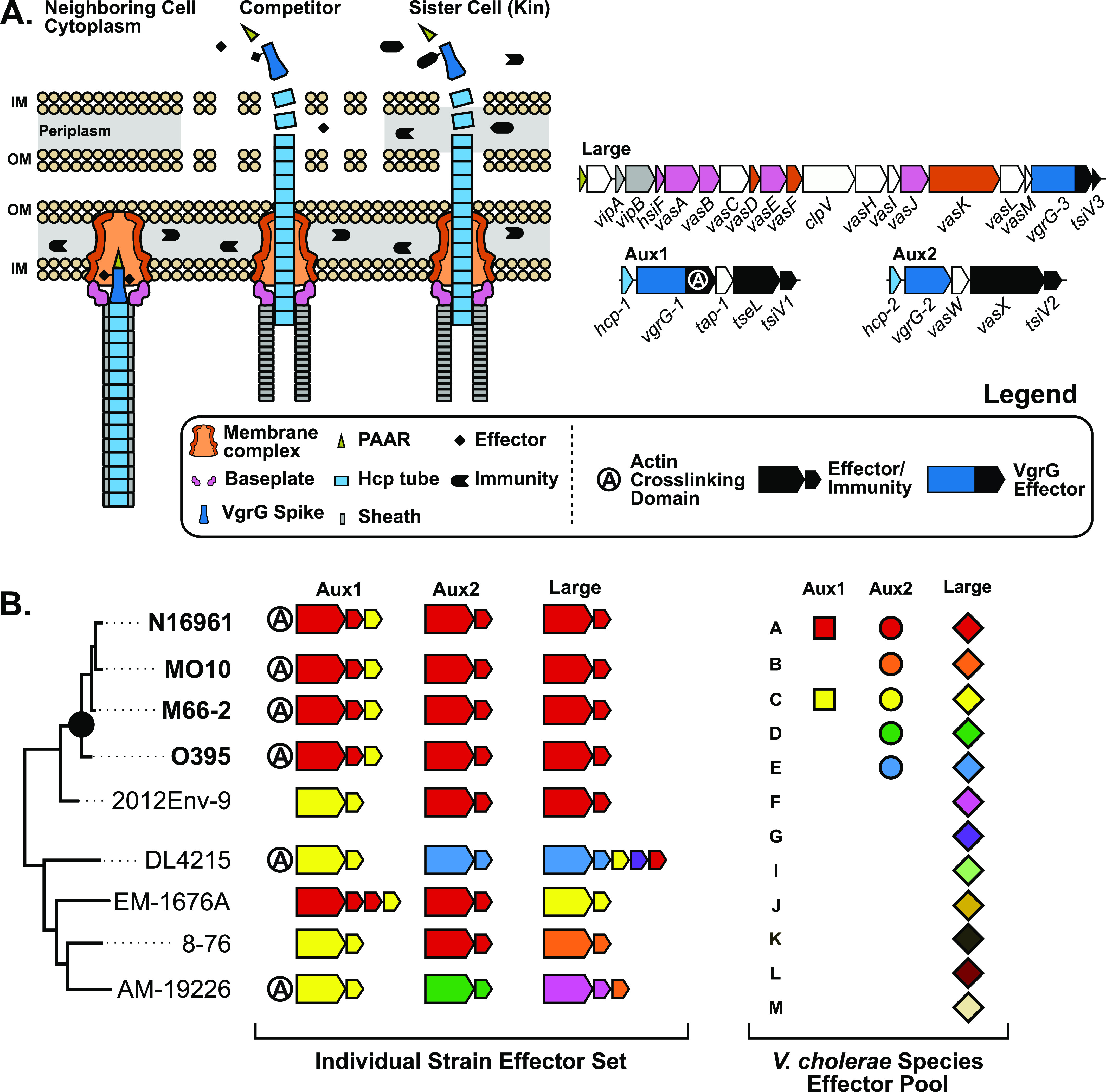
Pandemic *V. cholerae* strains encode a defined T6SS effector set. **(A)** Left: schematic of the T6SS in its extended and contracted states in competition with a neighboring non-kin or kin cell. Right: diagrams of the core *V. cholerae* T6SS loci. Genes are colored by their corresponding T6SS component in the schematic to the left (white coding regions are not represented). **(B)** Left: *V. cholerae* effector types and their distribution across a hypothetical *V. cholerae* phylogenetic tree. The pandemic clade of *V. cholerae* is indicated by a black dot, and individual strains within the clade are shown in bold. Effector and immunity genes present at each T6SS locus within each strain are shown to the right. Effector-immunity pairs are colored according to their type. Extra immunity genes without a neighboring effector gene indicate orphan immunity genes. Right: All known effector types found across the three core T6SS loci. Different shapes for each locus indicate that effector-immunity pairs of the same type at different loci are distinct proteins (ex: Aux1 A = lipase, Aux2 A = pore forming toxin).

Bacteria live in constant contact with shifting populations of bacterial and eukaryotic competitors. Survival often depends on the acquisition of defense systems like the Type VI Secretion System (T6SS), a harpoon-like nanomachine encoded by 25% of gram-negative bacteria (Pukatzki et al, 2006; Bingle et al, 2008; Boyer et al, 2009). The T6SS is evolutionarily related to the T4 phage contractile tail (Pukatzki et al, 2007; Leiman et al, 2009; Pell et al, 2009; Basler et al, 2012) and is used for contact-dependent translocation of toxic effector proteins into neighboring bacterial and eukaryotic cells (Pukatzki et al, 2006; Ma et al, 2009; MacIntyre et al, 2010) (Fig 1A). For bactericidal effectors, the effector-secreting cell also encodes a cognate immunity protein to protect against attacks from its kin cells (Hood et al, 2010; Russell et al, 2011; Brooks et al, 2013; Dong et al, 2013; Fritsch et al, 2013; Miyata et al, 2013). The T6SS was first identified in *Vibrio cholerae* (Pukatzki et al, 2006) and is highly conserved in the *Vibrio* genus (Weber et al, 2009; Yu et al, 2012; Church et al, 2016; Tang et al, 2016; Huang et al, 2017; Kirchberger et al, 2017).

All *V. cholerae* strains encode the T6SS in three genetic clusters (Pukatzki et al, 2009; Unterweger et al, 2014) (Fig 1A). The large cluster encodes most of the structural components of the system, including the inner membrane anchoring complex, the baseplate complex from which extends the contractile sheath, and two proteins involved in sheath extension and firing (Zoued et al, 2014; Cianfanelli et al, 2016; Schneider et al, 2019) (Fig 1A). The large cluster also encodes a VgrG spike (*vgrG-3*) with a bactericidal C-terminus and its cognate immunity factor (*tsiV3*) (Brooks et al, 2013; Dong et al, 2013). The two remaining clusters, termed auxiliary clusters 1 and 2 (Aux1 and Aux2), each encode an Hcp protein that forms the inner tube atop which VgrG spikes are loaded, an alternate VgrG spike, a chaperone protein for effector loading (Unterweger et al, 2015), and a distinct effector-immunity pair. In a subset of *V. cholerae* strains, the Aux1 VgrG-1 protein is sometimes fused to a C-terminal actin crosslinking domain with anti-eukaryotic properties (Pukatzki et al, 2007) (Fig 1A). In addition to the three conserved loci, four additional T6SS-associated genetic elements (Aux3-6) are found sporadically across *V. cholerae* strains (Altindis et al, 2015; Labbate et al, 2016; Crisan et al, 2019; Drebes Dörr & Blokesch, 2020). This work will focus on the three core loci: Aux1, Aux2, and the large cluster.

Strains of the *V. cholerae* species fall along a spectrum of virulence to humans, including harmless environmental strains, opportunistic pathogens, and pathogens that have adapted to the human gastrointestinal tract (Faruque et al, 2004). Once ingested, some pathogenic *V. cholerae* strains cause deadly secretory diarrhea known as cholera. Most of these strains belong to the pandemic-generating lineage, a monophyletic group of strains descended from an ancestor with the O1 serogroup (Islam et al, 2017) (Fig 1B). Whereas the T6SS is conserved across *V. cholerae*, different strains encode different effectors (Unterweger et al, 2014; Kirchberger et al, 2017; Hussain et al, 2021). To date, two effector types have been identified at Aux1 (A or C), five effector types have been identified at Aux2 (A–E), and 12 effector types have been identified at the large cluster (A–G, I–M). It is important to note that different effector genes at Aux1 and Aux2 encode distinct proteins, whereas variable types at the large cluster are different C-terminal extensions on a conserved VgrG spike (Unterweger et al, 2014) (Fig 1A). There is no cross-protection between types, and incongruence between effector sets can lead to inter- and intraspecies competition (Unterweger et al, 2014; Hussain et al, 2021). Environmental *V. cholerae* strains encode various different sets of effector types across the three T6SS loci (ex: Aux1 C/Aux2 D/Large F or CDF for short) (Fig 1B). In contrast, *V. cholerae* strains in the pandemic-generating lineage all encode an identical set of three distinct effectors referred to as A-type (*tseL*, *vasX*, *vgrG-3*, or AAA) and the *vgrG-1* actin crosslinking domain (Unterweger et al, 2014; Kirchberger et al, 2017) (Fig 1B). Considering this conservation of the AAA effector set and that the strain of *V. cholerae* that founded the pandemic-generating lineage likely had a constitutively active T6SS (Drebes Dörr et al, 2022), it is possible that acquisition of the AAA effector set was important for establishment of the pandemic-generating lineage and the eventual evolution of pandemic *V. cholerae*.

Not only are environmental *V. cholerae* T6SS effector sets highly variable, but T6SS effectors are horizontally transferred (Kirchberger et al, 2017; Thomas et al, 2017). One indication of effector exchange is the maintenance of past immunity genes without their cognate effectors (orphans) (Kirchberger et al, 2017) (Fig 1B). Whereas the recombination steps leading to orphan retention are unknown, we hypothesize that it is an evolutionary mechanism that allows a T6SS-producing strain to exchange effector-immunity types without becoming vulnerable to nearby ex-kin cells. Orphan immunity genes can be leveraged to investigate the evolutionary history of the T6SS in a given *Vibrio* strain. The Aux1 locus of pandemic *V. cholerae* encodes an orphan C-type immunity gene downstream from its A-type effector-immunity pair (Fig 1B). We hypothesize that a pre-pandemic strain of *V. cholerae* with an Aux1 C-type effector-immunity pair (CAA) acquired the Aux1 A-type effector-immunity pair (*tseL/tsiV1*) by horizontal gene transfer, giving rise to the AAA effector set conserved across the pandemic clade. *V. cholerae* strains resembling these CAA ex-kin cells make up a sister clade to pandemic *V. cholerae* (Fig 1B, strain 2012Env-9).

This study investigated the evolutionary history of the pandemic *V. cholerae*–associated T6SS effectors, with the goal of identifying the donor of the Aux1 A-type effector-immunity module to the *V. cholerae* pandemic-generating lineage. Our search for pandemic-associated T6SS effectors across the *Vibrio* genus indicated the fish pathogen *V. anguillarum* as a potential reservoir of A-type effectors. We showed that all currently available strains of *V. anguillarum* encode the Aux1 A-type effector *tseL*, and many strains also encode *vasX*, *vgrG-3*, and the *vgrG-1* actin crosslinking domain. We bioinformatically assessed the prevalence and directionality of transfer events of T6SS effectors between the two species. Finally, we demonstrated experimentally that the *tseL-tsiV1* effector-immunity pair from *V. cholerae* and the homologous A-type pair from *V. anguillarum* cross-neutralize each other, indicating selective pressure to defend against attacks from the opposite species. Together, our findings suggest that acquiring T6SS effectors from the aquatic pathogen *V. anguillarum* may have helped a pre-pandemic *V. cholerae* strain gain a competitive advantage in the aquatic reservoir along its path to founding the pandemic *V. cholerae* clade.

## Results

### Cross-species effector gene distribution indicates the presence of pandemic-associated *V. cholerae* T6SS effectors in a clade of fish-colonizing *Vibrio* species

**Figure S1.**
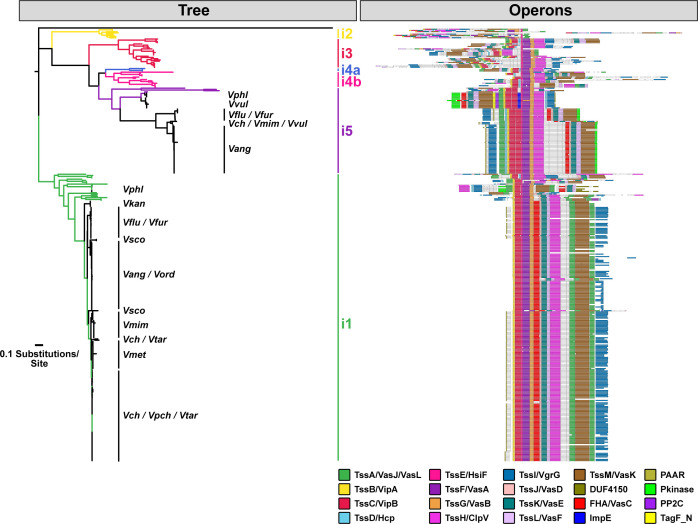
T6SS^i^ large cluster subtype distribution across select *Vibrio* species. Hamburger (https://github.com/djw533/hamburger) analysis of all analyzed *Vibrio* genomes showing all identified complete T6SS clusters. Grouping of the identified T6SS clusters with known T6SS types is shown by the phylogenetic tree (left), and operon structure is indicated (right). Colored branches of the tree indicate Hamburger-internal representative T6SSs corresponding to the indicated type, and black branches indicate input genomes. Operon diagrams are centered around *vipA*/*vipB*. *V. cholerae = Vch*, *V. paracholerae* = *Vpch*, *V. tarriae = Vtar*, *V. metoecus* = *Vmet*, *V. mimicus* = *Vmim*, *V. fluvialis* = *Vflu*, *V. furnissii* = *Vfur*, *V. anguillarum* = *Vang*, *V. ordalii* = *Vord*, *V. vulnificus* = *Vvul*, *V. parahaemolyticus* = *Vphl*, *V. scophthalmi = Vsco*, *V. kanaloae* = *Vkan*.

**Figure S2.**
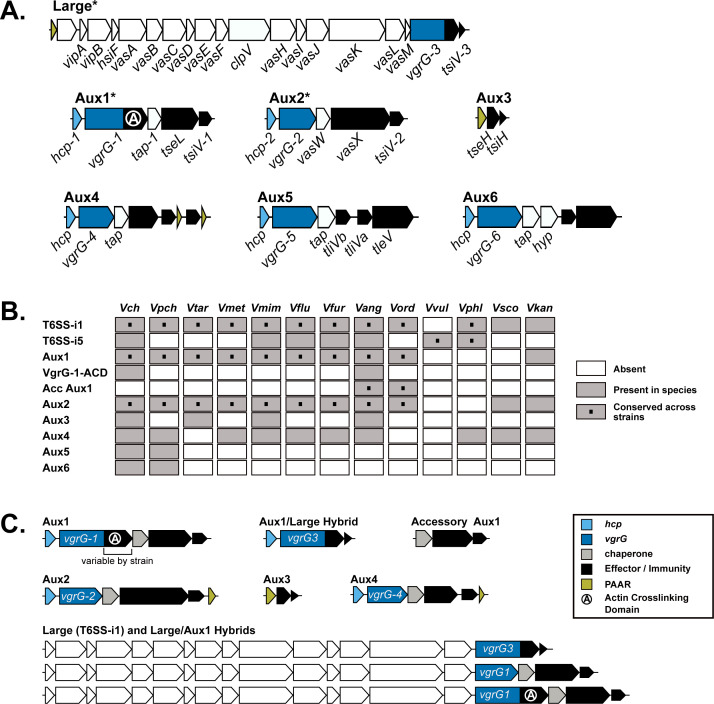
*V. anguillarum* encodes most known *V. cholerae* T6SS loci and a single-T6SS cluster unique to *V. anguillarum* and *V. ordalii*. **(A)** Diagrams of known *V. cholerae* T6SS loci. Coding regions are colored according to the legend in (C). Core T6SS loci (conserved across all *V. cholerae* strains) are marked with an *. **(B)** Heat map of all T6SS clusters in *V. cholerae* and *V. anguillarum* and their distribution across all analyzed *Vibrio* species. *V. cholerae = Vch*, *V. paracholerae* = *Vpch*, *V. tarriae = Vtar*, *V. metoecus* = *Vmet*, *V. mimicus* = *Vmim*, *V. fluvialis* = *Vflu*, *V. furnissii* = *Vfur*, *V. anguillarum* = *Vang*, *V. ordalii* = *Vord*, *V. vulnificus* = *Vvul*, *V. parahaemolyticus* = *Vphl*, *V. scophthalmi = Vsco*, *V. kanaloae* = *Vkan*. **(C)** Schematic diagrams of T6SS clusters identified in *V. anguillarum*, including the unique hybrid clusters and Accessory Aux1.

To assess the distribution of pandemic *V. cholerae* T6SS effector proteins across *Vibrio* species, we first compiled a database of 247 isolates across 14 *Vibrio* species and identified the T6SS clusters encoded within their genomes (Figs S1 and S2). Whereas the T6SS is functionally conserved across gram-negative species, the core components of the T6SS vary. T6SSs can be phylogenetically classified into four types (T6SS^i^, T6SS^ii^, T6SS^iii^, and T6SS^iv^) based on multiple independent acquisition events from phages (Bingle et al, 2008; Boyer et al, 2009; Barret et al, 2013; Russell et al, 2014; Böck et al, 2017). The canonical T6SS^i^ large cluster found in *Vibrio* species and other Proteobacteria can be further divided into six subtypes (1, 2, 3, 4a/b, and 5) based on T6SS structural gene content and organization (Boyer et al, 2009; Barret et al, 2013). Our search identified a single T6SS^i1^cluster in all species but *V. vulnificus*, which encoded only a T6SS^i5^. Some strains of *V. parahaemolyticus*, *V. fluvialis*, *V. furnissii*, *V. anguillarum*, *V. cholerae*, and *V. mimicus* also encoded a T6SS^i5^ in addition to their T6SS^i1^ (Figs S1 and S2B and Table S1). All analyzed *V. vulnificus* strains encode a T6SS^i5^ similar to the T6SS^i5^ locus in *V. parahaemolyticus*, whereas two *V. vulnificus* strains carry an extra T6SS^i5^ with homology to the T6SS^i5^ found in *V. fluvialis*, *V. furnissii*, *V. anguillarum*, *V. cholerae*, and *V. mimicus* (Fig S1 and Table S1). Based on the reported dispersion of the latter T6SS^i5^ in *V. vulnificus* (López-Pérez et al, 2019) and our cross-species analysis, this entire locus is likely horizontally transferred.

Table S1 Large T6SS gene clusters identified in all strains.

Next, we focused on the auxiliary clusters (containing some but not all elements of a functional T6SS) in each species. To date, six auxiliary clusters (Aux1-6) have been identified in *V. cholerae* (Fig S2A). In our dataset, the Aux5 and Aux6 clusters were unique to *V. cholerae* and *V. paracholerae* (Fig S2B). Our analysis corroborated and expanded upon previous studies to show that the *V. cholerae* Aux1, Aux2, and large loci are conserved across *V. paracholerae*, *V. tarriae*, *V. metoecus*, “*V. parilis*” (not validly published), *V. mimicus*, *V. fluvialis*, and *V. furnissii* species (Huang et al, 2017; Kirchberger et al, 2017; Hussain et al, 2021) (Fig S2B). Our analyses also demonstrated that these core T6SS loci are conserved across all analyzed strains of *V. anguillarum* and *V. ordalii* – two *Vibrio* species that are commonly isolated from the sea water, sediment, phytoplankton, and aquaculture settings (Fig S2B and C and Table S2). In most of the 63 *V. anguillarum* genomes, a region homologous to the *V. cholerae* Aux1 cluster from *vgrG-1* through *tsiV1* is encoded at the end of the large cluster, and a *vgrG-3* homolog is encoded next to the *hcp* gene usually associated with Aux1 (Fig S2C). These hybrid clusters were likely produced by recombination between the conserved 5′-regions of *vgrG-1* in the Aux1 cluster and *vgrG-3* in the large cluster. The *V. anguillarum* species also encoded Aux3 (2 of 63) and Aux4 (18 of 63). A subset of *V. anguillarum* strains causes a fatal hemorrhagic septicemia, termed vibriosis, in over 50 species of fresh and saltwater fish and, less commonly, mollusks and crustaceans (Frans et al, 2011).

Table S2 Auxiliary T6SS gene clusters identified in *V. anguillarum* strains.

**Figure 2.**
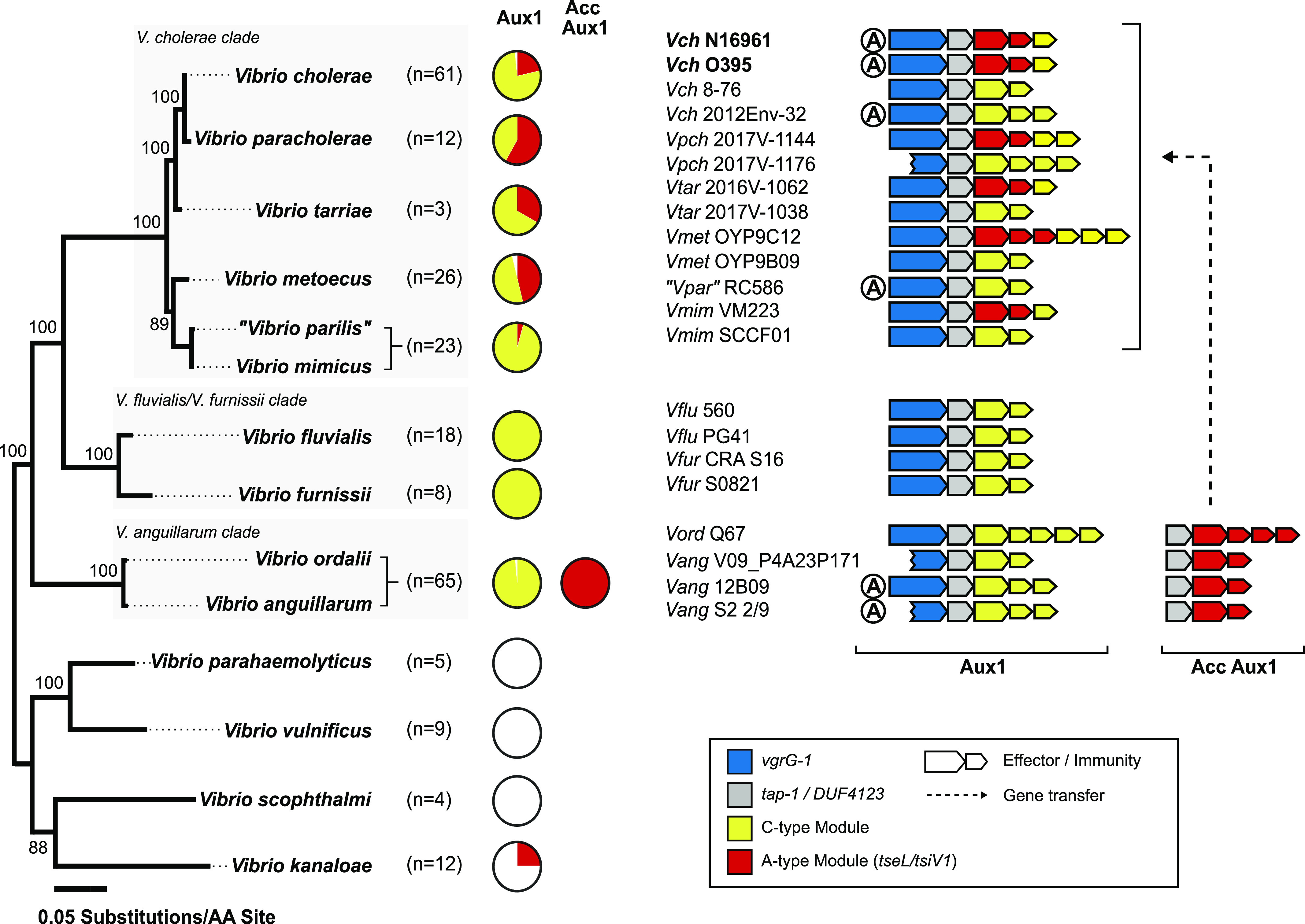
*V. cholerae* and *V. anguillarum* share the T6SS Aux1 A-type effector. Left: Maximum-likelihood tree built on 30 *Vibrio* genomes collapsed by species. Bootstrap support values are indicated. Scale represents substitutions per amino acid site. Pie charts showing the distribution of *V. cholerae* Aux1 A- and C-type effectors in each species. The novel *V. anguillarum/V. ordalii* T6SS cluster Acc Aux1 is included in the pie charts as it uniformly encodes the *V. cholerae* Aux1 A-type effector. Individual strain effector sets are shown in the expanded phylogenetic trees for each species in the supplement (Figs S3 and S4). Right: Diagrams of representative Aux1 clusters in the *V. cholerae* clade, the *V. fluvialis/V. furnissii* clade, and the *V. anguillarum* clade as well as the Acc Aux1 cluster found in the *V. anguillarum* clade. Putative gene transfer event of the A-type effector-immunity module between the *V. anguillarum* clade and the *V. cholerae* clade is indicated by a dashed arrow. Pandemic *V. cholerae* strains are shown in bold.

**Figure S3.**
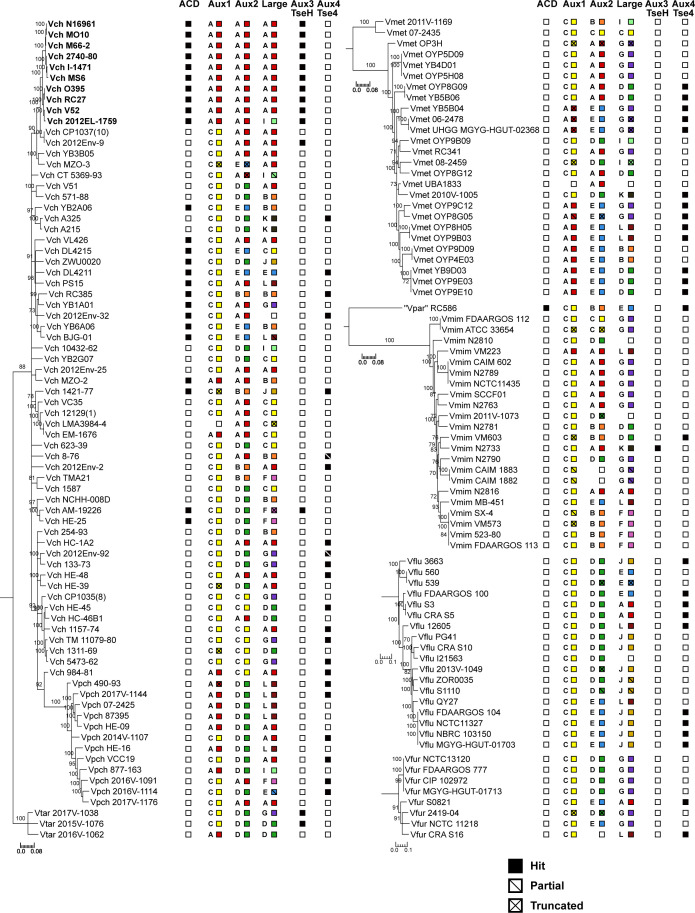
T6SS cluster and effector/immunity type distribution across *Vch*, *Vpch*, *Vtar*, *Vmet*, *“Vpar”*, *Vmim*, *Vflu*, and *Vfur* strains. Species trees for *Vch*, *Vpch*, *Vtar*, *Vmet*, “*Vpar*”, *Vmim*, *Vflu*, and *Vfur*. One maximum-likelihood tree based on core-genome SNP sites for each species or group of species. Bootstrapping support values are shown next to corresponding nodes. All nodes with bootstrapping support values below 70 are collapsed. Columns next to trees represent T6SS effectors at different clusters from the *Vch* population. Filled boxes represent presence. Empty boxes represent absence. Slashes indicate partial or truncated hits. Both letter and corresponding color (Fig 1B) of the present effector type are shown for effectors with multiple types. All scale bars represent substitutions/SNP site. Pandemic *V. cholerae* strains are shown in bold.

**Figure S4.**
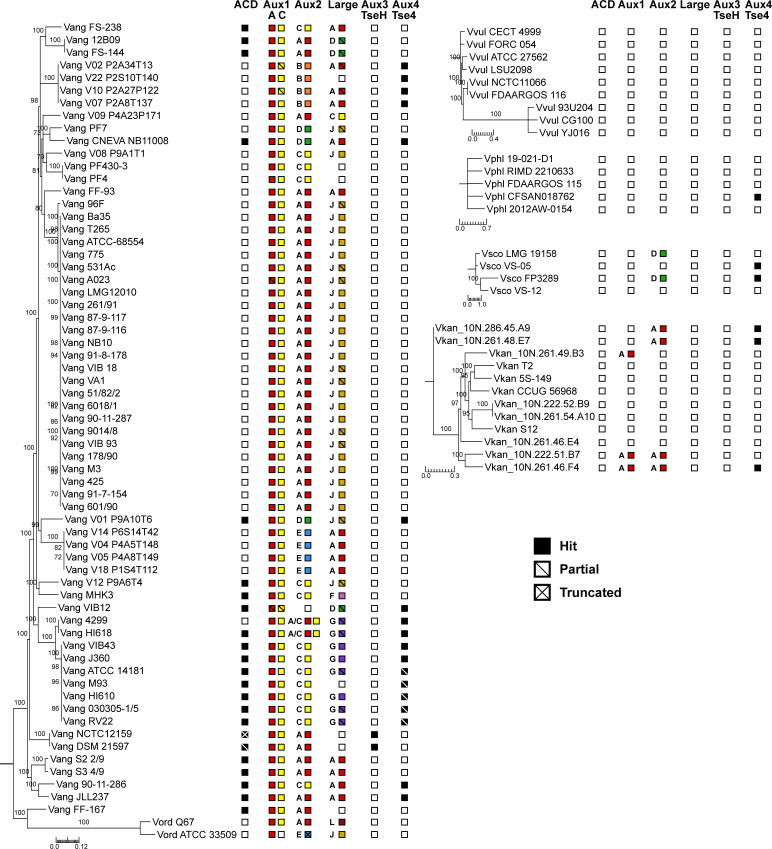
T6SS cluster and effector/immunity type distribution across *Vang*, *Vord*, *Vvul*, *Vphl*, *Vsco*, and *Vkan strains*. Species trees for *Vang*, *Vord*, *Vvul*, *Vphl*, *Vsco*, and *Vkan*. One maximum-likelihood tree based on core-genome SNP sites for each species. Bootstrapping support values are shown next to corresponding nodes. All nodes with bootstrapping support values below 70 are collapsed. Columns next to trees represent T6SS effectors at different clusters from the *Vch* population. Filled boxes represent presence. Empty boxes represent absence. Slashes indicate partial or truncated hits. Both letter and corresponding color (Fig 1B) of the present effector type are shown for effectors with multiple types. All scale bars represent substitutions/SNP site.

Because of the similarity in T6SS loci, we next assessed the relationship of the *V. anguillarum/V. ordalii* (*V. anguillarum* clade) genomes in our dataset to *V. cholerae*/*V. paracholerae*/*V. tarriae/V. metoecus*/“*V. parilis*”/*V. mimicus* (*V. cholerae* clade). We first constructed a multi-species, core-genome phylogeny based on 959 proteins shared across the 14 *Vibrio* species (Fig 2). The *V. anguillarum* clade branches from a common ancestor that established the *V. cholerae* and *V. fluvialis/V. furnissii* clades, indicating that *V. anguillarum* and *V. ordalii* are more distant relatives of *V. cholerae* than are *V. fluvialis* and *V. furnissii*. We next assessed the distribution of known *V. cholerae* effector types (Fig 1B) across the selected *Vibrio* species (Figs S3 and S4). The distribution of effector types at the Aux1 cluster (Fig 2) indicates that the C effector-immunity pair is widespread across the *V. cholerae*, *V. fluvialis/V. furnissii*, and *V. anguillarum* clades and was likely encoded by the founding Aux1 cluster in a common ancestor that pre-dates the formation of the three clades. In contrast, the Aux1 A effector (*tseL*) is irregularly distributed across the three clades, indicating possible horizontal transfer of this effector-immunity module (Fig 2). Previous work has demonstrated that the Aux1 A effector is highly enriched in the pandemic clade of *V. cholerae* and is sporadically distributed throughout environmental *V. cholerae*, *V. paracholerae*, *V. metoecus*, and *V. mimicus* strains (Kirchberger et al, 2017). We show for the first time that the Aux1 A effector is widespread within the *V. anguillarum* clade (Fig 2). Interestingly, *V. anguillarum* and *V. ordalii* uniformly encode both Aux1 A and C effectors because of the presence of both a standard Aux1 cluster and a newly identified cluster in each genome (Figs S2 and S4). This T6SS cluster was absent from all other analyzed species. This cluster lacked *hcp* and *vgrG* genes, encoding only a putative DUF4123-containing T6SS chaperone protein (Unterweger et al, 2015), an effector gene, and anywhere from one to seven immunity genes (Figs 2 and S2). As this cluster always encodes an Aux1 A-type effector homologous to *V. cholerae* TseL (Figs 2 and S4), we have named it Accessory Aux1 (Acc Aux1). In all cases, the *V. anguillarum* Aux1 cluster encoded a C-type effector, and the Acc Aux1 cluster encoded an A-type effector (Figs 2 and S4). Like the Aux1 A-type effector *tseL*, all other pandemic-associated *V. cholerae* effectors, except for the large cluster A-type effector *vgrG-3*, were found to be restricted to the *V. cholerae* and *V. anguillarum* clades. The *vgrG-1* actin crosslinking domain is only observed in *V. cholerae*, “*V. parilis*,” and the *V. anguillarum* clade (Figs S3 and S4). Because of the presence of the Aux1 A effector (*tseL*) in a novel T6SS gene cluster widespread in the *V. anguillarum* clade, we focused on the evolutionary history of this effector gene.

### Aux1 A-type T6SS effectors are horizontally transferred between *V. anguillarum* and *V. cholerae*

**Figure S5.**
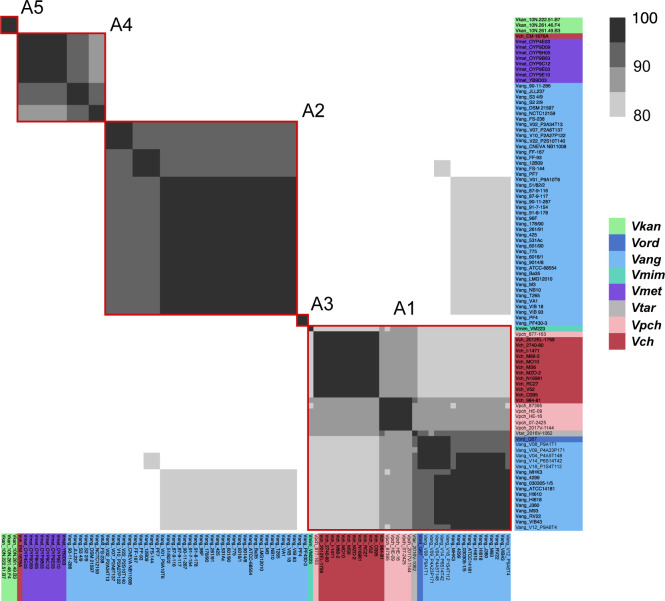
Aux1 A-type effector proteins are divided into subtypes. Pairwise amino acid identity heat map of Aux1 A effector proteins (TseL) from *Vch*, *Vpch*, *Vtar*, *Vmet*, *Vmim*, *Vang*, *Vord*, and *Vkan*. Heat map is restricted to 80–100% identity. Red boxes indicate distinct clusters called by CD-HIT with an 80% identity cutoff.

The Aux1 A-type effector distribution suggests that it was shared horizontally between *V. anguillarum* and *V. cholerae*, as the A-type effector is distributed sporadically across the *Vibrio* phylogenetic tree and is restricted to the *V. cholerae* and *V. anguillarum* clades (Figs 2, S3, and S4). Furthermore, every identified A-type Aux1 cluster in the *V. cholerae* clade also carries at least one C-type orphan immunity gene, indicating that these strains are derived from one or more Aux1 C ancestors, and the Aux1 A-type effector was acquired from outside of the *V. cholerae* clade (Figs 1B and 2). The *V. anguillarum* clade is of particular interest as an Aux1 A donor due to the presence of the Acc Aux1 cluster, which does not encode any C-type orphans and likely encodes an A-type effector ancestrally. Thus, we aimed to identify transfer events between the *V. cholerae* clade and the *V. anguillarum* clade. To identify potential donor Acc Aux1 clusters, we extracted all identified A-type effector and immunity genes from each species and clustered the corresponding amino acid sequences with an 80% cutoff. From this analysis, we grouped the Aux1 A effector into five subtypes (A1–A5) (Fig S5). Every strain in the *V. anguillarum* clade carried a single A-type effector at the Acc Aux1 cluster that grouped with subtypes A1–A4. Aux1 subtypes A2 and A3 are unique to the *V. anguillarum* clade, whereas A1 and A4 are shared between the *V. cholerae* and *V. anguillarum* clades. This finding indicates that the A-type effector in the *V. anguillarum* clade was inherited vertically and diverged along with the divergence of *V. anguillarum* strains, but the sporadically distributed A-type effectors in the *V. cholerae* clade were acquired horizontally from *V. anguillarum*.

**Figure S6.**
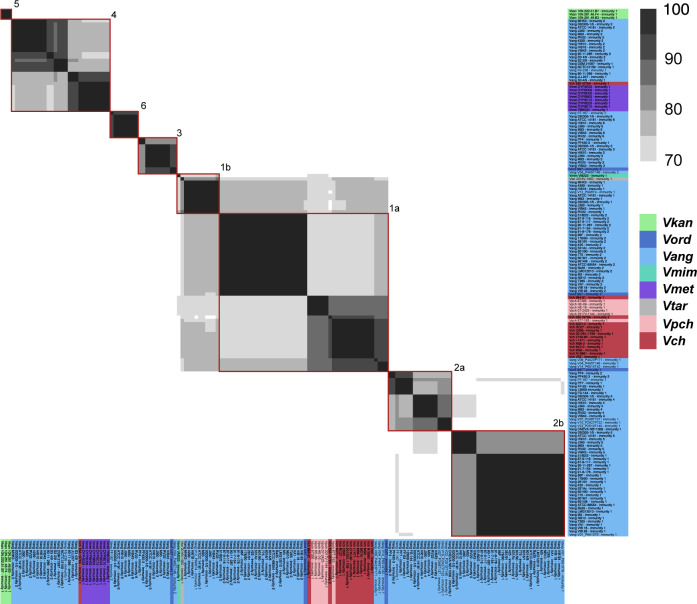
Aux1 A-type immunity proteins are further divided beyond subtype. Pairwise amino acid identity heat map of Aux1 A immunity proteins (TsiV1) from *Vch*, *Vpch*, *Vtar*, *Vmet*, *Vmim*, *Vang*, *Vord*, and *V. kan*. Heat map is restricted to 70–100% identity. Red boxes indicate distinct clusters called by CD-HIT with a 70% identity cutoff.

**Figure 3.**
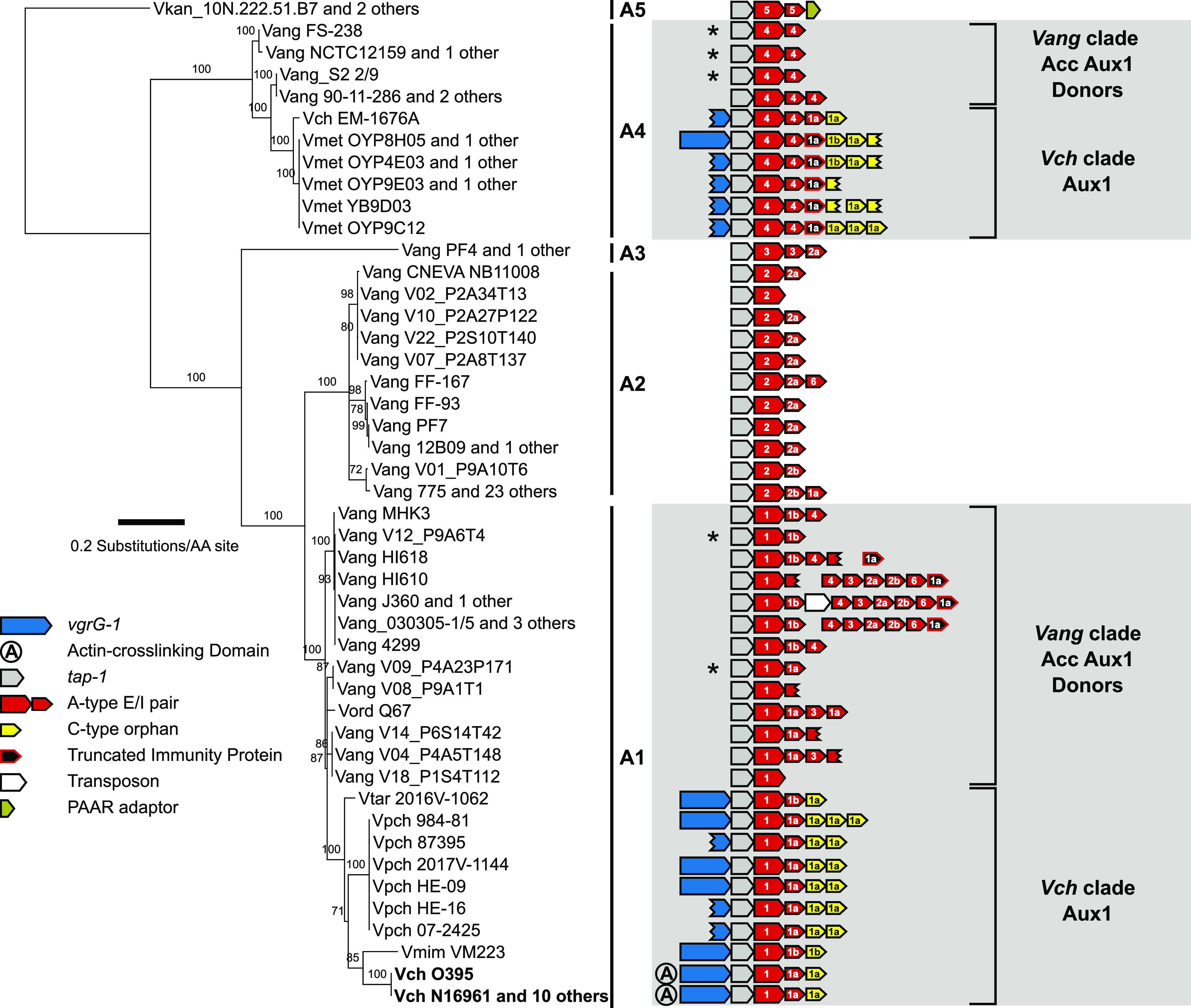
*V. anguillarum* Acc Aux1 was horizontally transferred to *V. cholerae*. Maximum-likelihood tree of Aux1 A-type effectors (TseL). Support values for each node are shown. Scale represents substitutions per amino acid site. Acc Aux1 and/or Aux1 diagram for each strain is shown on the right, with effector-immunity type and subtype indicated by color and labels. Grey areas indicate groups of strains potentially involved in the transfer of the A-type effector from *V. anguillarum* to *V. cholerae*. Asterisks mark the *V. anguillarum* Acc Aux1 clusters most likely transferred to *V. cholerae*. Pandemic *V. cholerae* strains are shown in bold.

Next, we aimed to increase the resolution of our gene transfer observations by subtyping the Aux1 A immunity genes. The Aux1 A immunity genes are more diverse on the amino acid level than their cognate effectors (Fig S6), so we lowered our clustering cutoff to 70%. The resulting clusters lead us to subdivide Aux1 A1 and A2 into A1a/b and A2a/b. We next built a single-protein phylogeny of the Aux1 A effectors and overlaid the corresponding Aux1 or Acc Aux1 clusters for each strain along with their effector-immunity subtypes (Fig 3). From this tree, it is evident that the Aux1 A1a effector-immunity pair found in *V. cholerae* and *V. paracholerae* strains most likely originated from a group of *V. anguillarum* and *V. ordalii* strains (*V. anguillarum* V09_P4A23P171, *V. anguillarum* V04_P4A5T148, *V. anguillarum* V14_P6S14T42, and *V. ordalii* Q67) that encode A1a immunity genes ranging from 89.8–91.1% nucleotide identity and 91.7–92.5% amino acid identity to the pandemic *V. cholerae* A1a immunity gene (Fig S6). We cannot determine the terminal donor of the Aux1 A1a effector-immunity pair to the *V. cholerae* pandemic clade, as *V. paracholerae* strains also encode an Aux1 A1a effector-immunity pair with 91.7% nucleotide identity and 89.5% amino acid identity to the pandemic *V. cholerae* A1a immunity gene (Fig S6). One *V. paracholerae* strain (877-163) even encodes an Aux1 A1a effector-immunity pair nearly identical to the pandemic *V. cholerae* Aux1 locus (Fig S6). Regardless, the presence of orphan C immunity genes at all *V. paracholerae* Aux1 clusters (Fig 3) supports *V. anguillarum* as the initial donor to the *V. cholerae* clade. Beyond the Aux1 A1a transfer event, there were at least two more transfers of Aux1 A effector-immunity pairs between the *V. anguillarum* and *V. cholerae* clades. All *V. metoecus* strains with an Aux1 A effector and *V. cholerae* EM-1676A encode an A4 effector-immunity pair, most likely originating from a different sub-clade of *V. anguillarum*, including strains S2 2/9, NCTC12159, and FS-238 (Fig 3). The Aux1 A1b effector found in *V. tarriae* 2016V-1062 and *V. mimicus* VM223 may have originated from a group of several A1b-encoding *V. anguillarum* strains, including V12_P9A6T4 (Fig 3).

### Recombination between *V. cholerae* Aux1 and Acc Aux1 occurred at least twice

**Figure S7.**
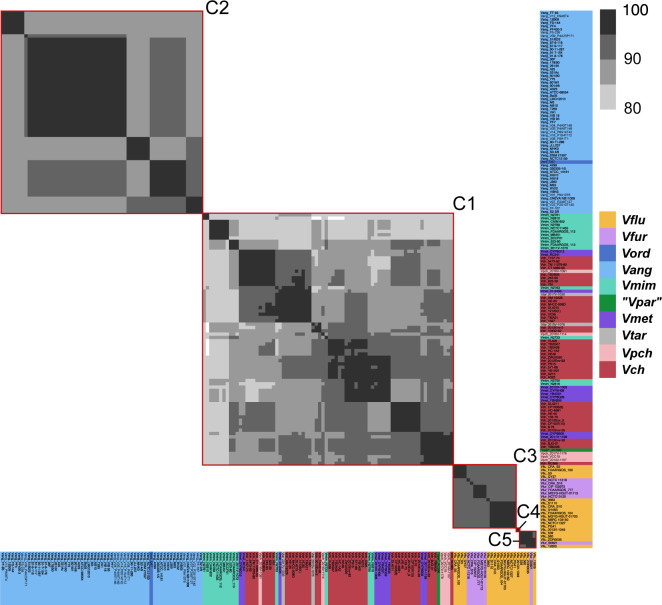
Aux1 C-type effector proteins are divided into subtypes. Pairwise amino acid identity heat map of Aux1 C effector proteins from *Vch*, *Vpch*, *Vtar*, *Vmet*, “*Vpar*”, *Vmim*, *Vang*, *Vord*, *Vfur*, and *Vflu*. Heat map is restricted to 80–100% identity. Red boxes indicate distinct clusters called by CD-HIT with an 80% identity cutoff.

**Figure S8.**
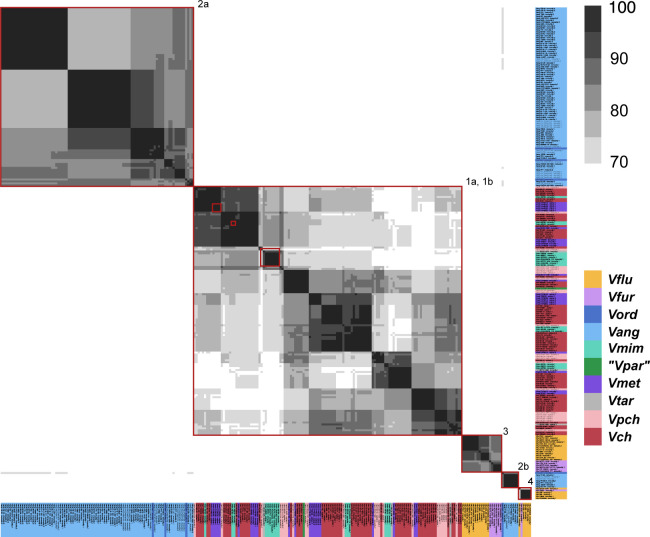
Aux1 C-type immunity proteins are further divided beyond subtype. Pairwise amino acid identity heat map of Aux1 C immunity proteins from *Vch*, *Vpch*, *Vtar*, *Vmet*, “*Vpar*”, *Vmim*, *Vang*, *Vord*, *Vfur*, and *Vflu*. Heat map is restricted to 70–100% identity. Red boxes indicate distinct clusters called by CD-HIT with a 70% identity cutoff.

**Figure 4.**
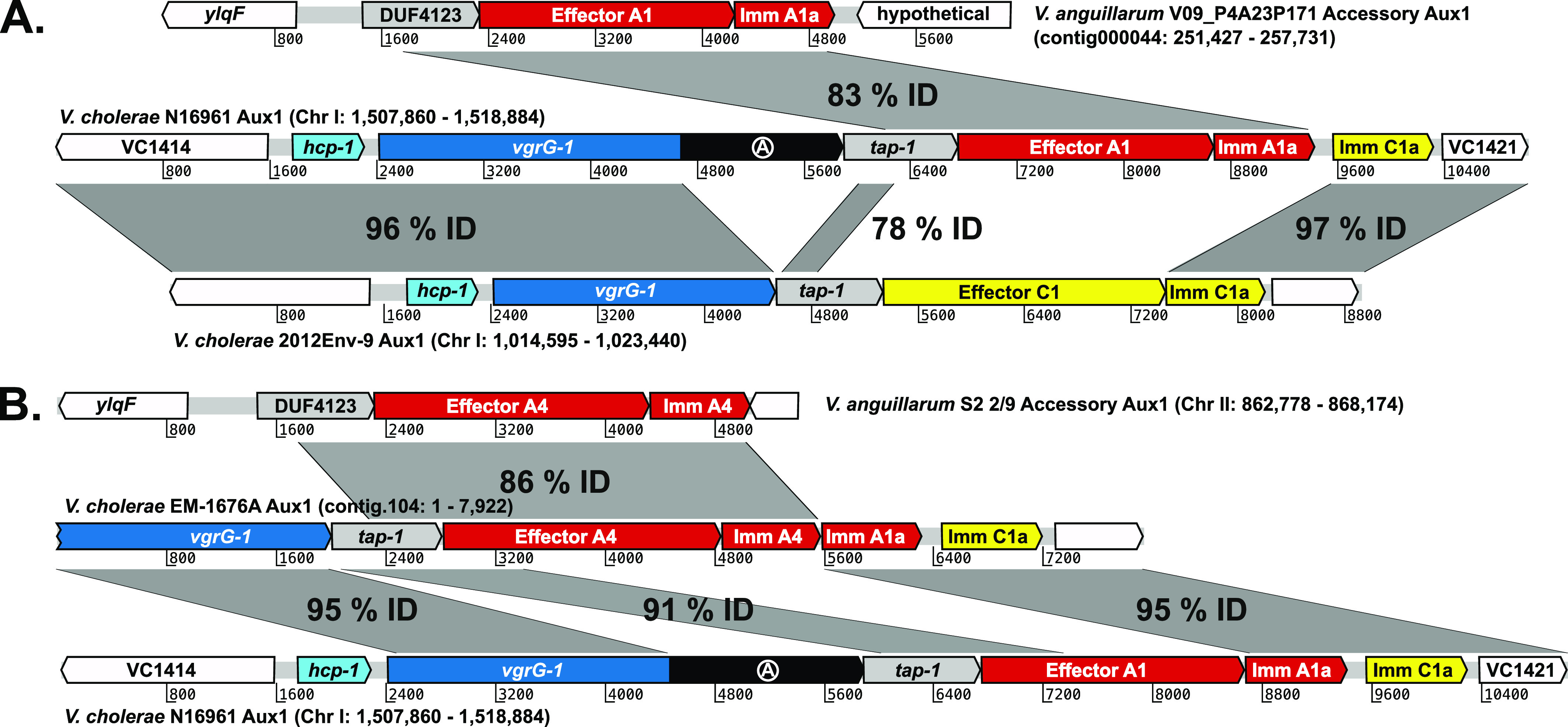
A-type effectors from *V. anguillarum* recombined into the *V. cholerae* Aux1 locus at least twice. **(A)** Artemis alignment of the T6SS Aux1 cluster from pandemic *V. cholerae* strain N16961 with its potential Acc Aux1 donor (top) and Aux1 recipient (bottom) clusters, representing the transfer event indicated in Fig 3 (bottom grey box). **(B)** Alignment of the T6SS Aux1 cluster from *V. cholerae* strain EM-1676A with its potential Acc Aux1 donor (top) and Aux1 recipient (bottom), representing the transfer event indicated in Fig 3 (top grey box). Homologous regions are shown by grey boxes labeled with the nucleotide identity.

**Figure S9.**
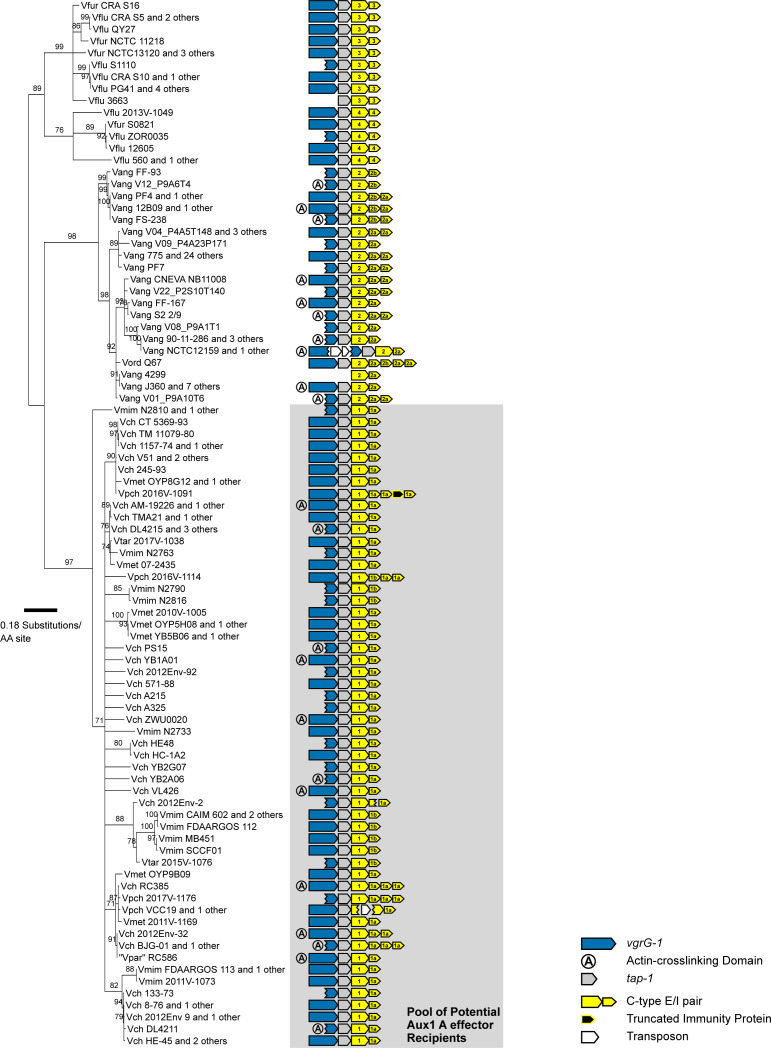
Aux1 C effector/Immunity subtypes are not horizontally transferred between clades. Maximum-likelihood tree of identified Aux1 C-type effectors from strains lacking an Acc Aux1 cluster or an A-type effector at Aux1. Bootstrapping support values for each node are shown. Scale bar represents substitutions per amino acid site. Aux1 schematic for each strain is shown on the right. Each effector/immunity cassette is labeled with its associated subtype. A grey box indicates a group of potential recipient strains involved in the transfer of the A-type effector from *Vang* and the generation of an orphan immunity gene in *Vch*.

Next, we used the Aux1 C-type orphan immunity genes present in the *V. cholerae* clade to identify strains that constitute likely recipients of the Aux1 A effector-immunity module from the *V. anguillarum* clade. To identify potential Aux1 recipient clusters, we extracted all identified C-type effector and immunity genes, including bona fide (encoded next to their cognate effector) and orphan immunity genes, from each species and clustered the corresponding amino acid sequences with a cutoff of 80%. Like the A-type effectors, we identified five C effector subtypes (C1–C5) (Fig S7). Again, the cognate immunity genes for two C subtypes were more variable than their effectors, leading to further subdivisions (C1a/b and C2a/b) (Fig S8). Unlike the Aux1 A effectors, C effector subtypes were clade-specific, with C1 restricted to the *V. cholerae* clade, C2 restricted to the *V. anguillarum* clade, and C3–5 restricted to the *V. fluvialis/V. furnissii* clade, indicating that the C effector was acquired by a common ancestor of the clades and diverged along with speciation. We next overlaid our Aux1 A effector phylogeny with our newly established Aux1 C effector-immunity subtypes (Fig 3). Each orphan-encoding Aux1 A cluster in the *V. cholerae* clade carries a C1 type orphan. Based on this result, we concluded that on one or more occasions, the Acc Aux1 cluster from a *V. anguillarum* strain was transferred to the *V. cholerae* clade, where it recombined with an Aux1 C1 cluster (Figs 4 and S9). An alternate scenario in which Acc Aux1 recombined with the Aux1 C2 cluster in the *V. anguillarum* clade to create the orphan-encoding locus we see in *V. cholerae* is unlikely based on the distribution of orphan C immunity subtypes.

The *V. cholerae* 2012Env-9 Aux1 cluster is a potential predecessor of the pandemic Aux1 locus. It encodes a bona fide C immunity protein >95% identical to the orphan C immunity protein encoded by pandemic strains (Fig 4A). *V. cholerae* 2012Env-9 is also a member of a sister clade to the *V. cholerae* pandemic clade (Fig 1B) and is thus a close extant relative of the pre-pandemic ancestor that received the Aux1 A effector (Kirchberger et al, 2017). Recombination between Acc Aux1 and Aux1 likely occurred by homology-facilitated illegitimate recombination, an interspecies gene transfer mechanism (de Vries & Wackernagel, 2002; Prudhomme et al, 2002; Meier & Wackernagel, 2003; Amarir-Bouhram et al, 2011). A crossover occurred in a region of strong homology between the Acc Aux1 DUF4123-containing chaperone and the Aux1 *tap-1* chaperone. The second crossover event likely occurred between the 3′ end of the donor A1 immunity gene and the 3′ end of the C1 effector, despite no apparent homology between the two genes, leading to the displacement of the C1 effector and maintenance of the C1 orphan (Fig 4A). All Aux1 A4–encoding *V. metoecus* strains and *V. cholerae* EM-1676A encode an A1 type orphan as well as the C1 orphan (Fig 3). This array of orphan immunity genes indicates that the transfer of the Acc Aux1 A4 effector-immunity pair into *V. metoecus/V. cholerae* was likely a second independent transfer event in which the incoming Acc Aux1 A4 cluster recombined into an Aux1 cluster that had already acquired the Aux1 A1 effector-immunity pair (Fig 4B). There is no obvious recipient Aux1 cluster into which Acc Aux1 A4 recombined to form the *V. cholerae* EM-1676A Aux1. The *V. cholerae* EM-1676A Aux1 cluster encodes type C1a and A1a orphan immunity genes but no *vgrG-1* actin crosslinking domain (Figs 3 and 4B). Based on this, we hypothesize that Acc Aux1 A4 either recombined into an Aux1 cluster with a matching immunity gene array but no actin crosslinking domain or a pandemic-like Aux1 that later lost its actin crosslinking domain. Clusters backing either hypothesis, while likely extant, are not represented in our dataset.

### *V. cholerae* Aux1 A1a and *V. anguillarum* Acc Aux1 A1a effector-immunity pairs cross-neutralize

**Figure 5.**
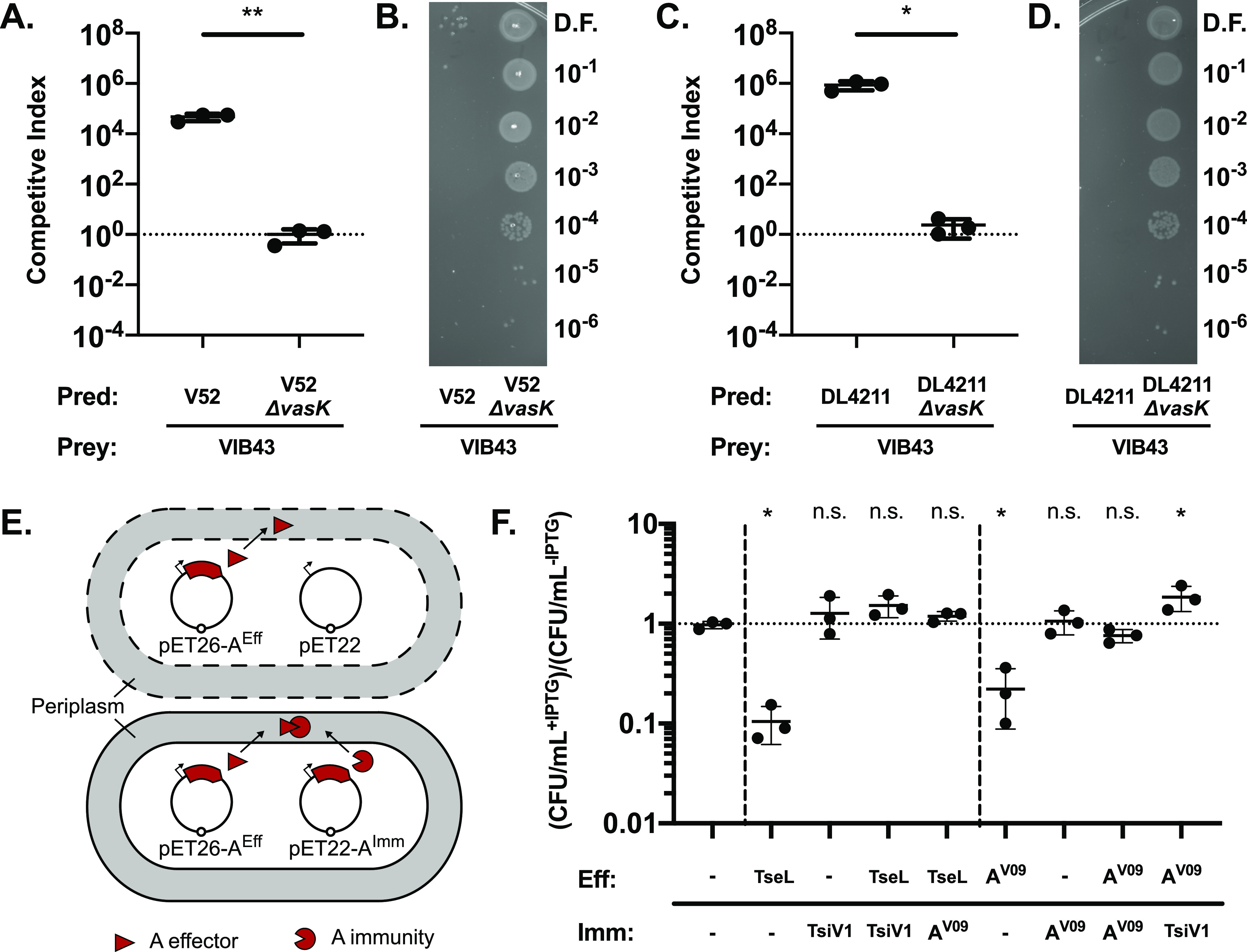
A-type effector-immunity pairs from *V. cholerae* Aux1 and *V. anguillarum* Acc Aux1 are cross-neutralizing. **(A)** Killing assays for *V. cholerae* strain V52 or V52 Δ*vasK* versus *V. anguillarum* strain VIB43. **(B)** Dilution spots of VIB43 prey cells from killing assays in (A). **(C)** Killing assay for *V. cholerae* strain DL4211 or DL4211 Δ*vasK* versus *V. anguillarum* strain VIB43. **(D)** Dilution spots of VIB43 prey cells from killing assays in (C). **(A, C)** Significance was determined by unpaired *t* test (*P = 0.0115, **P = 0.0056). **(E)** Diagram of *E. coli* dual-expression viability assay. Dashed cell membranes represent lysis. **(F)** Viability assay with results plotted as the ratio of CFU/ml recovered with induction (+IPTG) to CFU/ml recovered without induction (−IPTG). TseL/TsiV1 = *V. cholerae* A1 effector and A1a immunity protein. A^V09^ = *V. anguillarum* A1 effector or A1a immunity protein. Significance was determined by one-way ANOVA with Dunnet’s multiple comparisons test (*P = [0.0190, 0.0486, 0.0182], n.s., non-significant). All comparisons were made to strain with two empty vectors. **(A, C, F)** Quantitative results are from three independent experiments (n = 3). Individual replicates are shown. Horizontal bars represent the mean, and error bars represent SD. Source data are available online for this figure.

It is possible that *V. cholerae* and *V. anguillarum* co-occupy a niche in the aquatic reservoir and compete in a T6SS-dependent manner, leading to a selective pressure to horizontally acquire and maintain immunity genes that can neutralize attacks from the opposing species (Hussain et al, 2021). First, we hypothesized that *V. cholerae* and *V. anguillarum* would exhibit a degree of cross-killing in a T6SS-dependent manner. Thus, we performed killing assays with two different strains of *V. cholerae* with constitutively active T6SSs, the O37 pathogenic strain V52 and the environmental strain DL4211, against a *V. anguillarum* isolate (VIB43). The T6SS effector set of *V. anguillarum* VIB43 is incompatible with V52 and DL4211 (Figs S3 and S4). After 4 h of co-incubation at 28°C on agar at a 1:1 ratio, both strains of *V. cholerae* outcompeted *V. anguillarum* VIB43 (Fig 5A–D). Importantly, co-incubation of *V. anguillarum* VIB43 with *V. cholerae* carrying an in-frame deletion in the T6SS membrane complex component *vasK* (V52 Δ*vasK* and DL4211 Δ*vasK*) shows no competitive advantage for either strain (Fig 5A–D). Based on these results, it is possible that the *V. anguillarum* T6SS is not active in strain VIB43 under our experimental conditions. In these killing assays, *V. cholerae* strain V52, which encodes the Aux1 A1a effector-immunity pair TseL-TsiV1 (Figs S5 and S6), exhibited 18-fold less killing of VIB43 than did DL4211, which encodes an Aux1 C1a effector-immunity pair (Figs S7 and S8). This result implies some degree of protection from T6SS killing in VIB43, which carries an Acc Aux1 A1a effector-immunity pair, when competed against the partially compatible strain V52 compared with the completely incompatible strain DL4211. Next, we hypothesized that if members of the *V. cholerae* pandemic clade and *V. anguillarum* regularly competed in the environment, then there would be a selective pressure for the *V. cholerae* Aux1 A1a effector-immunity pair to maintain its ability to neutralize the *V. anguillarum* Acc Aux1 A1a effector-immunity pair. This idea is supported by recent findings showing that even a single–amino acid change in a T6SS immunity gene is sufficient to lose protection against its cognate effector (Kostiuk et al, 2021). Regular T6SS-dependent competition between strains would likely constrain mutations within the effector and immunity genes to maintain protective capacity. To test our hypothesis, we co-expressed the A-type effector from *V. cholerae* N16961 (TseL) or *V. anguillarum* V09_P4A23P171 (Aeff^V09^), each fused to an N-terminal periplasmic secretion signal, with both their cognate immunity gene and the opposing immunity gene (TsiV1 or Aimm^V09^, respectively) in *Escherichia coli* (Fig 5E). These strains were chosen because *V. cholerae* N16961 is a pandemic strain, and *V. anguillarum* V09_P4A23P171 is the most likely donor of the A-type effector-immunity pair found in pandemic strains of *V. cholerae* (Figs 3 and 4A). Expressing either TseL or Aeff^V09^ alone leads to an ∼10-fold reduction in the number of viable cells, whereas expression of either TsiV1 or Aimm^V09^ alone did not affect cell viability (Fig 5F). Cells co-expressing TseL or Aeff^V09^ with their cognate immunity genes had no significant reduction in viability. Neither was a reduction in viability observed when the effectors were co-expressed with the opposing immunity gene. These results support our hypothesis that the *V. cholerae* Aux1 A1a effector-immunity pair and its homologous pair in the *V. anguillarum* Acc Aux1 locus were under selective pressure to maintain cross-neutralization despite only 92.2% amino acid identity between their immunity genes.

## Discussion

*V. cholerae* and *V. anguillarum* are aquatic organisms and may share common niches. The previous studies described below have demonstrated, both in vitro and in silico, that *V. anguillarum* and *V. cholerae* come into contact in the environment. It has been shown experimentally that *V. cholerae* can maintain conjugative R-plasmids from *V. anguillarum* (Nakajima et al, 1983), indicating that *V. cholerae* and *V. anguillarum* can share DNA. More recently, it was discovered that a genomic island in four strains of O1 El Tor *V. cholerae* is 97% identical at the nucleotide level to a homolog in *V. anguillarum* VIB43 (Morita et al, 2020). Similar loci have also been identified in multiple non-O1 *V. cholerae* strains (Nguyen et al, 2018). Our findings further support the idea that *V. cholerae* interacts with *V. anguillarum*, as we identify gene transfer events between the two species. Specifically, we have shown here that a pre-pandemic *V. cholerae* strain carrying an Aux1 C1 effector acquired the Aux1 A1 effector through past physical contact with *V. anguillarum*. We also show that *V. cholerae* can efficiently kill *V. anguillarum* with its T6SS. The DNA released from lysed *V. anguillarum* cells can then be taken up by *V. cholerae* (Borgeaud et al, 2015; Veening & Blokesch, 2017). This is likely not a one-way interaction, as we show that *V. anguillarum* encodes its own arsenal of T6SS effectors. The *V. anguillarum* T6SS does not appear to be active in strain VIB43 under our experimental conditions, but the *V. anguillarum* T6SSs are active under conditions mimicking marine or intra-host conditions (Tang et al, 2016; Lages et al, 2019).

*V. cholerae* and *V. anguillarum* might interact in the gut and on the skin of fish. Both *V. anguillarum* and *V. cholerae* are chemotactic towards fish intestinal mucus, with *V. cholerae* showing an equivalent chemotactic response to both fish and human intestinal mucus (O’Toole et al, 1999). Furthermore, a sizeable metagenomic study of the mucosal skin surface of eels across multiple water sources showed that *Vibrio* species, including *V. anguillarum*, *V. cholerae*, *V. furnissii*, and *V. metoecus*, are highly concentrated in the skin mucus of estuarine eels (32% *Vibrio*) compared with the surrounding water (1% *Vibrio*) (Carda-Diéguez et al, 2017). Therefore, it is likely that the fish skin and gut concentrate interactions between *V. cholerae* and *V. anguillarum* and provide stable niches for interaction, interspecies killing, and gene transfer. Like *V. cholerae*, *V. anguillarum* is also isolated from sediment, phytoplankton, and zooplankton. Thus, the chitinous exoskeleton of zooplankton is also a potential niche for the interaction of *V. cholerae* and *V. anguillarum*, as *V. cholerae* chitin metabolism induces the T6SS and natural competence (Meibom, 2005; Borgeaud et al, 2015). Isolates of *V. anguillarum* have been shown to encode chitinase and grow with chitin and N-acetylglucosamine as their sole carbon sources (Hunt et al, 2008). Co-colonization of zooplankton by the two species could lead to bi-directional killing and genetic exchange. Our results do not favor either niche as the site of interaction and gene transfer between these two species.

*V. cholerae*, including some toxigenic O1 strains, has been isolated from 30 different species of fish (Halpern & Izhaki, 2017). Fish colonization by *V. cholerae* could provide benefits, such as protection from the aquatic environment and dissemination across long distances by fish harboring the bacteria and birds that eat the fish (Halpern et al, 2008; Laviad-Shitrit et al, 2019). *V. cholerae* can be isolated from several parts of the fish. The skin and intestinal mucosal surfaces are the most interesting of these for our study, as an adaptation to vertebrate mucus surfaces may have been necessary for the development of pathogenicity in humans. The zebrafish gut is a hospitable environment for *V. cholerae* (Runft et al, 2014; Mitchell et al, 2017). O1 El Tor infection of the zebrafish leads to sustained colonization, whereas infection with O1 classical strains or non-O1/non-O139 environmental strains leads to transient colonization (Runft et al, 2014; Mitchell et al, 2017). The T6SS could play a role in the sustained colonization of the fish gut, as O1 classical strains of *V. cholerae* carry an inactive T6SS (MacIntyre et al, 2010; Miyata et al, 2010; Kostiuk et al, 2021), and O1 El Tor strains should express their pathoadaptive T6SS in the fish gut. There are, however, several other differences between El Tor and classical *V. cholerae*, so there is likely more to fish colonization than simply having an active T6SS. Two studies have directly investigated the *V. cholerae* T6SS in the fish gut (Logan et al, 2018; Breen et al, 2021). Both studies show that the T6SS is unnecessary for initial colonization of the fish gut, but that T6SS expression can induce significant modulation of the fish gut microbiome. Little is known about the role of the T6SS in fish skin colonization. We believe that our results warrant further investigation of the role of the T6SS in fish colonization with a particular focus on different T6SS effector types, specifically pandemic-associated T6SS effectors like TseL. Improved colonization of the fish gut and skin could increase dissemination and potential adaptation to a host intestinal tract.

**Figure 6.**
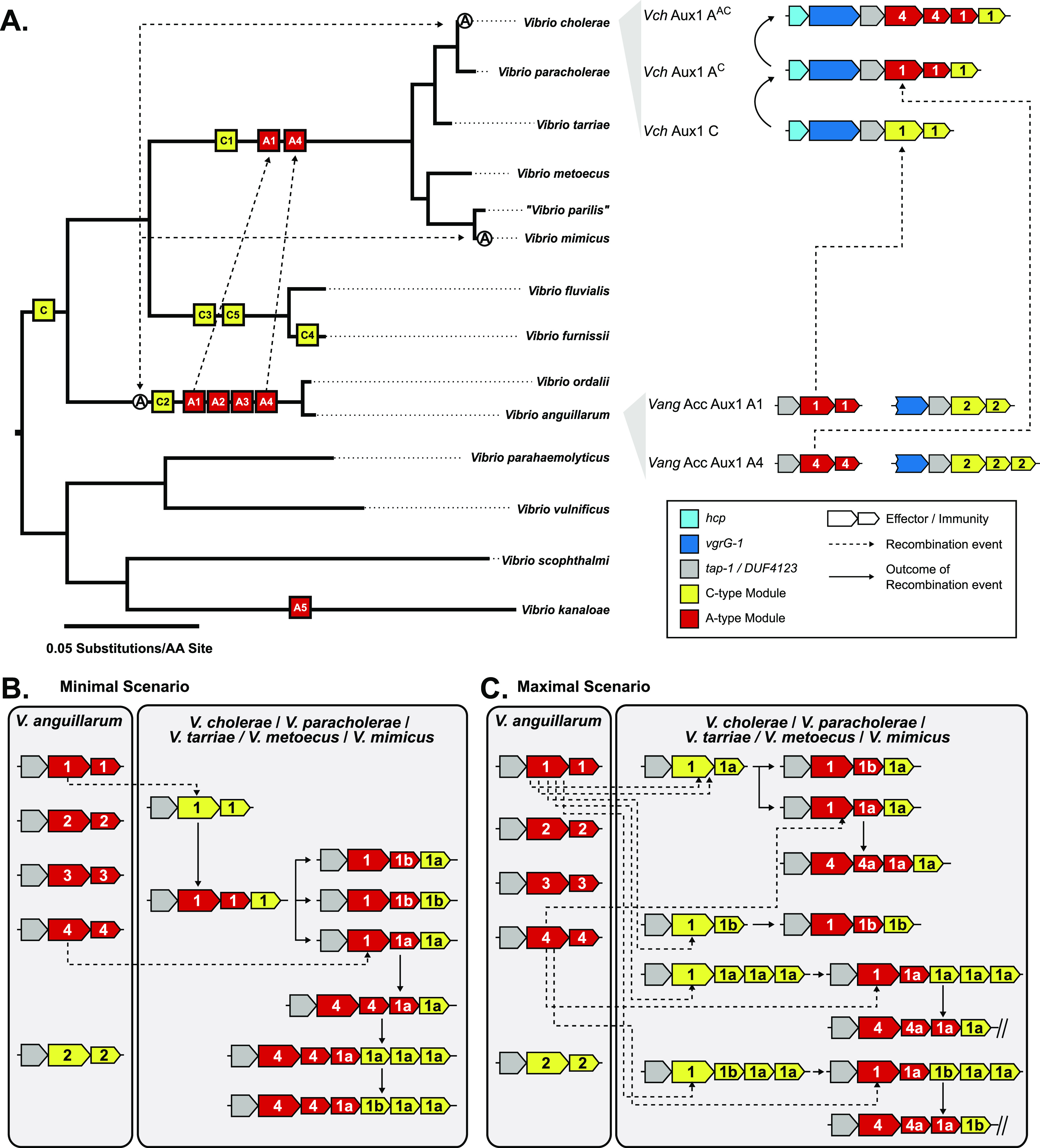
Model of pandemic-associated T6SS effector evolution at the Aux1 cluster of *V. cholerae*. **(A)** Left: Maximum-likelihood tree from Fig 3 overlaid with likely introduction points for each Aux1 effector type and the actin crosslinking domain of *vgrG-1*. Potential horizontal transfer events are indicated by uni- or bi-directional dashed arrows. Right: Representative genetic diagrams of Aux1 clusters in *V. cholerae* as well as Acc Aux1 and Aux1 clusters in *V. anguillarum*. **(B, C)** Schematic diagrams represent the potential transfer events leading to the array of Aux1 clusters observed in *V. cholerae*, *V. paracholerae*, *V. tarriae*, *V. metoecus*, and *V. mimicus*. **(B)** Minimal gene transfer scenario consisting of Aux1 A1 transfer, the divergence of the recipient cluster, subsequent transfer of Aux1 A4, and further divergence. **(C)** Maximal gene transfer scenario consisting of multiple individual transfer events for both Aux1 A1 and Aux1 A4. **(A, B, C)** Effector-immunity types are indicated by color, and effector-immunity subtypes are shown. Dashed arrows indicate recombination events, and solid arrows indicate the resulting cluster from said recombination events.

We showed that *V. cholerae* shares pandemic-associated T6SS effectors with *V. anguillarum*. Common T6SS effectors are likely an indication of a shared niche because acquiring effector-immunity sets similar to neighboring cells is a common protective strategy that is not unique to *V. cholerae* (Salomon et al, 2015; Steele et al, 2017; Thomas et al, 2017; Hussain et al, 2021). Within our dataset, the *V. cholerae* T6SS Aux1 A-type effector *tseL* is uniquely shared between the *V. cholerae* and *V. anguillarum* clades. It is important to note that *V. anguillarum* is more extensively sampled than *V. fluvialis/V. furnissii*, so we cannot rule out the presence of pandemic-associated T6SS effectors in this clade. Our data show that, on at least two occasions, A-type effectors originating in the *V. anguillarum* Acc Aux1 cluster were transferred to the *V. cholerae* clade (Fig 6). In the first transfer event, an A1a effector-immunity module was likely acquired by the *V. cholerae* pre-pandemic ancestor, displacing its C1 effector gene (Fig 6A). In the second, an A4 effector-immunity module replaced the A1 effector (Fig 6A). Based on the diversity of orphan C immunity arrays observed at Aux1 in the *V. cholerae* clade, it is likely that more than two transfers of the Aux1 A effector occurred between the two clades (Fig 6B and C). Our results show that an A1 effector protein closely related to the A1 effector of pandemic *V. cholerae* is found in approximately half of the analyzed *V. paracholerae* strains (Figs 3 and S3). As *V. cholerae* and *V. paracholerae* strains have been isolated from shared water sources (Islam et al, 2021), an alternate hypothesis to direct interaction between pre-pandemic *V. cholerae* and *V. anguillarum* is gene transfer between *V. anguillarum* and *V. paracholerae* and subsequent transfer to the *V. cholerae* pre-pandemic ancestor. There is currently no data, however, to support environmental interactions between *V. paracholerae* and *V. anguillarum*. Considering current environmental coincidence data, particularly in fish skin mucus, we believe that the most likely scenario is the direct transfer of the A1 allele of *tseL* from *V. anguillarum* to *V. cholerae*.

If *V. cholerae* shares niches and DNA, particularly Aux1 A T6SS effectors, with *V. anguillarum*, then this effector may be adaptive to a shared niche of *V. cholerae* and *V. anguillarum*. This raises the question of whether TseL plays an environmental role, as pandemic-associated *V. cholerae* genes have been shown to have ecological significance (Vezzulli et al, 2008). One potential ecological role of TseL could be fish colonization, which could somehow favor pandemicity. For instance, the VgrG-1 actin crosslinking domain has been shown to induce intestinal inflammation in the mouse infection model (Ma et al, 2009; Ma & Mekalanos, 2010) and peristalsis in the fish gut (Logan et al, 2018). This is a clear example of a T6SS effector beneficial for fish colonization and mammalian disease. Our results show that TseL is widespread in the *V. anguillarum* clade. Thus, we hypothesize that a parallel scenario may exist for TseL. We cannot link TseL to fish pathogenesis, as both pathogenic and non-pathogenic *V. anguillarum* isolates encode TseL, but it could play a role in colonization.

In this study, we assessed the distribution of T6SS effectors across 14 *Vibrio* species to reconstruct the evolutionary history of the pandemic *V. cholerae* T6SS and to indicate potential interspecies interactions. Our genomic results showed sharing of T6SS effector-immunity modules between *V. anguillarum* and *V. cholerae*. This led us to hypothesize that these two species may share one or more aquatic niches. We propose that *V. anguillarum* was the source of pandemic-associated *V. cholerae* T6SS effectors, indicating that the pandemic A-type effectors may play important roles outside human pathogenesis. Our findings highlight fish colonization as a potential stage during the evolution of pandemic *V. cholerae* and indicate that competitive fitness in the fish may have been necessary for pre-pandemic *V. cholerae* strains.

## Materials and Methods

### Acquisition and annotation of publicly available genome sequences

To select strains for a reduced database of *Vibrio* species, we first looked to identify species that likely come into contact with *V. cholerae* in nature. For this purpose, we leveraged the *V. cholerae* T6SS Aux3 and Aux4 clusters. These T6SS clusters are carried on mobile genetic elements that can readily excise from and insert into their host genomes (Labbate et al, 2016; Santoriello et al, 2020). Furthermore, the effector genes in these clusters are tightly conserved across strains (Santoriello et al, 2020; Crisan et al, 2021). The amino acid sequences of the Aux3 and Aux4 effectors (TseH and TpeV) (Table S3) were searched by BLASTp across all *Vibrio* species in the NCBI non-redundant protein database. This search returned hits from 11 different *Vibrio* species: *V. cholerae*, *V. metoecus*, *V. mimicus*, “*V. parilis*,” *V. fluvialis*, *V. furnissii*, *V. ordalii*, *V. anguillarum*, *V. scophthalmi*, *V. parahaemolyticus*, and *V. kanaloae*. The following steps were taken to compile a database of genomes:

(1) A selection of 76 *V. cholerae* genomes was chosen to include representatives from the pandemic clade, the closely related sister group, and diverse environmental strains. During the preparation of the manuscript, *V. paracholerae* and *V. tarriae* were introduced as newly defined species, the strains of which were previously denoted as *V. cholerae* or *Vibrio* species (Islam et al, 2021, 2022 *Preprint*). We found that 12 of our genomes originally annotated *V. cholerae* were members of the *V. paracholerae* species, and their names were changed to reflect this re-classification. Similarly, three genomes originally annotated as *Vibrio* species were members of the *V. tarriae* species, and their names were changed to reflect this re-classification.
(2) All available genomes at the time of database compilation were included for the following species: *V. metoecus*, *V. mimicus*, “*V. parilis*,” *V. fluvialis*, *V. furnissii*, *V. ordalii*, *V. anguillarum*, *V. scophthalmi*, and *V. kanaloae*.
(3) A selection of nine *V. vulnificus* and five *V. parahaemolyticus* genomes were included in the dataset as these species are prominent human pathogens like *V. cholerae*. The selected strains include *V. vulnificus* biotype 1 strain ATCC 27562, which is the most common *V. vulnificus* biotype and causes the bulk of human infections (Thiaville et al, 2011), and *V. parahaemolyticus* O3:K6 strain RIMD 2210633, the type strain of the clonal O3:K6 pandemic clade of *V. parahaemolyticus* (Ceccarelli et al, 2013).
(4) Finally, a BLASTp search was performed for the pandemic-associated *V. cholerae* Aux1 A-type effector (TseL, WP_000376836.1) in the NCBI non-redundant protein database. At the time of database construction, all species encoding a TseL homolog (>40% AA identity across >90% of the length of the protein) fell within the species that made up our database. Thus, we moved forward with our compiled database of 247 *Vibrio* genomes.

Table S3 Representative *V. cholerae* effector types.

Genome sequences used in this study are available from the NCBI GenBank database. Accession numbers for all strains are listed in Table S4. A single fasta file was generated for each genome, and all files were re-annotated with Prokka (v1.12) (Seemann, 2014). Re-annotation produced both GenBank and GFF files for each genome.

Table S4 *Vibrio* genomes analyzed in this study.

### Identification of T6SS clusters

Complete T6SS large clusters were extracted from generated GFF files using HMmer Based UndeRstandinG of gene clustERs (hamburger) (https://github.com/djw533/hamburger) with the -t option. Hamburger aligned the identified T6SS clusters with an internal reference set of T6SS cluster types (T6SS^i1^–T6SS^i5^) and built a phylogenetic tree of T6SS loci with corresponding gene diagrams aligned around the *vipA/vipB* cassettes. All identified T6SS large clusters are listed in Table S1. Hamburger does not identify Auxiliary T6SS clusters or effector and immunity genes. These were identified manually from our database of 247 genomes using the following three methods:

(1) *Vibrio* auxiliary clusters often encode *vgrG* genes, so auxiliary clusters were identified in an unbiased manner by performing a tBLASTn search for *vgrG-2* (VCA0018, WP_000212125.1) from *V. cholerae* N16961 with a Geneious grade cutoff of 60%. Geneious grade is a weighted metric combining query coverage (0.50), e-value (0.25), and pairwise identity (0.25). Grade is calculated using the following formula:
Grade = 50 ∗ fractionCoverage + 25 ∗ (*maximum*[0, 1 − eValue/10−20]) + 25 ∗ (*maximum*[0,[percentIdentity − minGradedIdentity]/[100 − minGradedIdentity]])
In this formula, minGradedIdentity is 50 for nucleotide and 25 for protein sequences. By using Grade, the longest, highest identity hits are prioritized, making it a much stronger metric than identity alone.
(2) Auxiliary clusters were identified in a biased manner by performing a tBLASTn search for all known T6SS effector types from *V. cholerae* (Table S3) with a Geneious grade cutoff of 30% for non-VgrG effectors and a cutoff of 60% for the VgrG effectors of the large cluster (Altindis et al, 2015; Labbate et al, 2016; Kirchberger et al, 2017; Crisan et al, 2019; Drebes Dörr & Blokesch, 2020). The N-terminal VgrG portion of all *V. cholerae* large cluster effector types is highly conserved and thus requires a higher cutoff to differentiate.
(3) Once auxiliary loci had been identified in each species, flanking genes were used to find missing loci in other strains of that species. Identified loci were extracted for downstream analyses. As this study focuses on *V. anguillarum*, the genomic locus of all identified T6SS auxiliary clusters from *V. anguillarum* strains are listed in Table S2.

### Core-genome and single-protein phylogeny

For Figs 3 and 6A: the set of core proteins encoded within the genomes of 30 *Vibrio* strains (Table S5) corresponding to 13 described species, including *V. paracholerae* and *V. tarriae*, two new species/subspecies closely related to *V. cholerae*, was extracted using usearch with an amino acid identity cutoff of 30% and then aligned using MUSCLE (Edgar, 2004, 2010), as implemented by the BPGA pipeline (Chaudhari et al, 2016). The resulting alignment of 959 core proteins, corresponding to 18,510 amino acid positions, was then used to construct a phylogenetic tree using the GAMMA + WAG substitution model in RAxML (v8.0.26) (Stamatakis, 2014). Branch support was calculated using 100 bootstrap replicates.

Table S5 *Vibrio* genomes used for the multi-species tree (Figs 3 and 6A).

For Figs S3 and S4: annotated GFF3 files were generated by Prokka (v1.12) (Seemann, 2014) and used for this analysis. Core genes were extracted from the GFF3 files based on their translated amino acid sequence using a cd-hit cutoff for homologous proteins of 95%, and extracted genes were aligned using Roary (v3.11.2) (Page et al, 2015). The core-genome alignment was reduced to only loci harboring polymorphisms using SNP sites (v2.4.1) (Page et al, 2016). A maximum-likelihood phylogenetic tree was built using RAxML (Stamatakis, 2014) with the GTR + GAMMA model. Statistical branch support was obtained from 100 bootstrap repeats.

Single-protein phylogenetic trees were constructed as follows: nucleotide sequences for genes of interest were extracted from annotated genomes and translated in Geneious (v2019.0.4). Only full-length sequences were used to generate single-protein phylogenetic trees. Any partial or truncated protein sequences were discarded. Protein sequences were aligned using MUSCLE (v3.8.425) (Edgar, 2004). Pairwise MUSCLE alignments were used for tree building with RAxML as described above. All phylogenetic trees were visualized with TreeGraph 2 (v2.15.0-887 β) (Stöver & Müller, 2010). Branches with bootstrapping support values <70 were collapsed.

### T6SS effector and immunity protein typing and subtyping

All sequence manipulations were performed in Geneious (v2019.0.4). All effector and immunity gene sequences were extracted from all identified Aux1 and Acc Aux1 clusters from all analyzed species. All effectors and immunity nucleotide sequences were translated. BLASTp was performed against a custom effector or immunity gene database generated by Kirchberger et al, (2017) for each amino acid sequence to identify effector or immunity type. Representative amino acid sequences used as BLASTp queries are shown in Table S3. For a given sequence, the strongest hit >30% identity was considered its type (Unterweger et al, 2014; Kirchberger et al, 2017). All effector or immunity amino acid sequences for a given type were aligned with MUSCLE (v3.8.425) (Edgar, 2004) to generate a pairwise amino acid identity matrix. Only full-length sequences were used. Clusters were determined by inputting protein fasta files to CD-HIT (Li & Godzik, 2006; Fu et al, 2012) with an 80% identity clustering cutoff for effector proteins and a 70% cutoff for immunity genes. Heat maps were made from pairwise matrices using pheatmap (pheatmap, R package pheatmap v1.0.12).

### Alignment of T6SS clusters

Full-length T6SS clusters were aligned using the Artemis Comparison Tool (ACT, v18.1.0) (Carver et al, 2005). Nucleotide sequences were submitted to NCBI BLASTn with the “align two or more sequences” option. Alignment files were exported from NCBI and input to ACT.

### Bacterial strains, plasmid construction, and growth conditions

*V. cholerae* strains, *V. anguillarum* strains, *E. coli* strains, plasmids, and primers used in this study are listed in Tables S6 and S7. *E. coli* strain DH5α λpir was used for cloning. All pET vectors were built with Gibson cloning. Vectors were cut with XhoI and BamHI. Effector or immunity genes were amplified with primers modified to place each gene in frame with the upstream start codon of the periplasmic localization signal sequence (pelB). All *E. coli* strains were routinely cultured at 37°C in Lysogeny Broth–Lennox (LB), shaking at 0.87*g*. Culture on agar plates was done on LB agar at 28°C (*V. cholerae*, *V. anguillarum*, and *E. coli*). When required, IPTG (for the induction of the T7 polymerase), NaCl (for *V. anguillarum* culture), or antibiotics were added to liquid or agar culture medium at the following concentrations: 100 µM IPTG, 2% wt/vol NaCl, 100 µg/ml streptomycin (Sm), 50 µg/ml rifampicin (Rif), 50 µg/ml kanamycin (Kan), 100 µg/ml ampicillin (Amp), and 30 µg/ml chloramphenicol (Cm).

Table S6 Bacterial strains and plasmids.

Table S7 Primers.

### Competitive killing assays

Predator *V. cholerae* (Sm^R^) and prey *V. anguillarum* (Rif^R^) strains were cultured on LB agar plates with 2% wt/vol NaCl and selective antibiotics. Plates were incubated for 18–20 h at 28°C. Predator and prey cells were collected from lawns with a sterile loop and resuspended in LB + 2% NaCl to an optical density at 600 nm (OD_600_) of 1. Input counts were determined by dilution plating of single-strain suspensions on LB agar + 2% NaCl with Rif or Sm. Predator and prey cells were mixed at a 1:1 ratio, and 25 µl of each mixture was spotted on LB agar + 2% NaCl. Spots were incubated for 4 h at 28°C. Spots were resuspended in 1 ml LB + 2% NaCl. Dilution series of output cell suspensions was plated on LB agar + 2% NaCl plates with Rif or Sm. Input and output plates were incubated for 18–20 h at 28°C. Input and output CFU counts were determined for predator and prey strains from each killing assay. Competitive indices were determined as follows: C.I. = ([Output CFU/mL^PREDATOR^/Output CFU/mL^PREY^]/[Input CFU/mL^PREDATOR^/Input CFU/mL^PREY^]).

### Effector-immunity co-expression viability assays

*E. coli* BL21 (DE3) pLysS (Cm^R^) strains carrying all pairwise combinations of vectors from the following two lists were generated: (1) pET26b, pET26b-tseL, or pET26b-Aeff^V09^ (Kan^R^) and (2) pET22b, pET22b-tsiV1, or pET22b-Aimm^V09^ (Amp^R^). Strains were grown (18–20 h) in 1 ml LB with selective antibiotics. Overnight cultures were normalized to an OD_600_ of 1 in fresh LB. Cell suspensions were spotted (10 µl) on LB agar + Cm + Kan + Amp both with and without 100 µM IPTG (inducing and non-inducing, respectively). Spot plates were incubated for 18–20 h at 28°C. Spots were collected by scraping and resuspended in 1 ml LB by vortexing. Output cell counts were determined by plating 10-fold serial dilutions of each cell suspension on LB agar + Cm + Kan + Amp. Output dilution plates were incubated for 18–20 h at 28°C. Viability indices were calculated as follows: Viability Index = ([Output CFU/ml^+IPTG^]/[Output CFU/ml ^−IPTG^]).

## Data Availability

Genome accession codes are in Table S4. Analyses were performed with publicly available tools referenced in the Methods and Supplemental Methods. Custom protein databases mentioned in the text are available upon request.

## Supplementary Material

Reviewer comments
