# Supplementary material for: Pandemic Vibrio cholerae acquired competitive traits from an environmental Vibrio species
Source: Life Sci Alliance. 2022 Nov 29;6(2):e202201437. doi: 10.26508/lsa.202201437 (PMC9711863; doi:10.26508/lsa.202201437)
Supplement: Supplementary file 1 [file LSA-2022-01437_TableS1.docx]

**Supplemental Table S1. Large T6SS gene clusters identified in all strains.**

| **Gene cluster/strain** | **contig** | **start** | **stop** | **T6SS subtype** |
| --- | --- | --- | --- | --- |
| Vang_030305-1_5_cluster_1 | RCUB01000046 | 7851 | 35904 | i5 |
| Vang_12B09_cluster_1 | AJYV02000004 | 777034 | 801172 | i1 |
| Vang_178_90_cluster_1 | CP011470 | 1522981 | 1551034 | i5 |
| Vang_178_90_cluster_2 | CP011471 | 606564 | 629172 | i1 |
| Vang_261_91_cluster_1 | CP010032 | 1522976 | 1551029 | i5 |
| Vang_261_91_cluster_2 | CP010033 | 609067 | 631675 | i1 |
| Vang_425_cluster_1 | CP020534 | 1444007 | 1472060 | i5 |
| Vang_425_cluster_2 | CP020533 | 855978 | 878586 | i1 |
| Vang_4299_cluster_1 | CP011458 | 1524454 | 1552507 | i5 |
| Vang_4299_cluster_2 | CP011459 | 610032 | 630530 | i1 |
| Vang_51_82_2_cluster_1 | CP010042 | 1522924 | 1550977 | i5 |
| Vang_51_82_2_cluster_2 | CP010043 | 606499 | 629107 | i1 |
| Vang_531Ac_cluster_1 | VSLE01000007 | 2476 | 30529 | i5 |
| Vang_531Ac_cluster_2 | VSLE01000047 | 2156 | 21253 | i1 |
| Vang_601_90_cluster_1 | CP010076 | 1522983 | 1551036 | i5 |
| Vang_601_90_cluster_2 | CP010077 | 606514 | 629122 | i1 |
| Vang_6018_1_cluster_1 | CP010291 | 1522922 | 1550975 | i5 |
| Vang_6018_1_cluster_2 | CP010292 | 606157 | 628765 | i1 |
| Vang_775_cluster_1 | CP002284 | 1524618 | 1552669 | i5 |
| Vang_775_cluster_2 | CP002285 | 611058 | 633666 | i1 |
| Vang_87-9-116_KU_cluster_1 | CP021980 | 1752652 | 1780705 | i5 |
| Vang_87-9-116_KU_cluster_2 | CP021981 | 73072 | 95680 | i1 |
| Vang_87-9-117_cluster_1 | CP010046 | 1522899 | 1550952 | i5 |
| Vang_87-9-117_cluster_2 | CP010047 | 604111 | 626719 | i1 |
| Vang_90-11-286_cluster_1 | CP011460 | 1680558 | 1708610 | i5 |
| Vang_90-11-286_cluster_2 | CP011461 | 778864 | 803002 | i1 |
| Vang_90-11-287_cluster_1 | CP011475 | 1523040 | 1551093 | i5 |
| Vang_90-11-287_cluster_2 | CP011476 | 606476 | 629084 | i1 |
| Vang_9014_8_cluster_1 | CP010038 | 1522994 | 1551047 | i5 |
| Vang_9014_8_cluster_2 | CP010039 | 605346 | 627954 | i1 |
| Vang_91-7-154_cluster_1 | CP010083 | 606638 | 629246 | i1 |
| Vang_91-7-154_cluster_2 | CP010082 | 1524072 | 1552125 | i5 |
| Vang_91-8-178_cluster_1 | CP010034 | 1522953 | 1551006 | i5 |
| Vang_91-8-178_cluster_2 | CP010035 | 609053 | 631661 | i1 |
| Vang_96F_cluster_1 | AEZA01000007 | 134397 | 154893 | i1 |
| Vang_96F_cluster_2 | AEZA01000016 | 470974 | 499027 | i5 |
| Vang_A023_cluster_1 | CP010036 | 1523020 | 1551073 | i5 |
| Vang_A023_cluster_2 | CP010037 | 609136 | 631744 | i1 |
| Vang_ATCC_14181_cluster_1 | MCJC01000022 | 147149 | 175202 | i5 |
| Vang_ATCC-68554_cluster_1 | CP023208 | 1297775 | 1325828 | i5 |
| Vang_ATCC-68554_cluster_2 | CP023209 | 897835 | 920443 | i1 |
| Vang_Ba35_cluster_1 | CP010030 | 1522918 | 1550971 | i5 |
| Vang_Ba35_cluster_2 | CP010031 | 609063 | 631671 | i1 |
| Vang_CNEVA_NB11008_cluster_1 | CP022103 | 1769059 | 1797120 | i5 |
| Vang_CNEVA_NB11008_cluster_2 | CP022104 | 72804 | 96972 | i1 |
| Vang_DSM_21597_cluster_1 | CP010084 | 1513139 | 1541192 | i5 |
| Vang_DSM_21597_cluster_2 | CP010085 | 641721 | 662218 | i1 |
| Vang_FF-167_cluster_1 | NZ_AJYR02000096 | 17345 | 40793 | i1 |
| Vang_FS-144_cluster_1 | AJYU02000212 | 26899 | 47396 | i1 |
| Vang_FS-238_cluster_1 | NZ_AJYS02000075 | 16332 | 40176 | i1 |
| Vang_HI610_cluster_1 | CP011462 | 1478791 | 1506844 | i5 |
| Vang_HI610_cluster_2 | CP011463 | 647971 | 668431 | i1 |
| Vang_HI618_cluster_1 | MNLD01000065 | 1510 | 20713 | i1 |
| Vang_J360_cluster_1 | CP034672 | 195605 | 223658 | i5 |
| Vang_J360_cluster_2 | CP034673 | 242864 | 267786 | i1 |
| Vang_JLL237_cluster_1 | NZ_CP022101 | 1740120 | 1768172 | i5 |
| Vang_JLL237_cluster_2 | NZ_CP022102 | 71832 | 95970 | i1 |
| Vang_LMG12010_cluster_1 | CP011468 | 1522933 | 1550986 | i5 |
| Vang_LMG12010_cluster_2 | CP011469 | 603958 | 626566 | i1 |
| Vang_M3_cluster_1 | CP006699 | 1524560 | 1552613 | i5 |
| Vang_M3_cluster_2 | CP006700 | 611037 | 633645 | i1 |
| Vang_M93_cluster_1 | NOWD01000014 | 134703 | 159937 | i5 |
| Vang_M93_cluster_2 | NOWD01000092 | 462 | 20450 | Undetermined |
| Vang_MHK3_cluster_1 | CP022469 | 240067 | 263491 | i1 |
| Vang_NB10_cluster_1 | LK021130 | 1746381 | 1774434 | i5 |
| Vang_NB10_cluster_2 | LK021129 | 74583 | 97191 | i1 |
| Vang_NCTC12159_cluster_1 | UGPJ01000001 | 3067484 | 3095537 | i5 |
| Vang_NCTC12159_cluster_2 | UGPJ01000002 | 263213 | 287078 | i1 |
| Vang_PF4_PUCV_cluster_1 | CP023290 | 777345 | 800058 | i1 |
| Vang_PF430-3_cluster_1 | CP011467 | 642342 | 665055 | i1 |
| Vang_PF7_cluster_1 | CP011465 | 262041 | 283452 | i1 |
| Vang_RV22_cluster_1 | AEZB01000085 | 2296 | 30351 | i5 |
| Vang_S2_2_9_cluster_1 | CP011473 | 593806 | 624628 | i1 |
| Vang_S3_4_9_cluster_1 | CP022100 | 71810 | 95948 | i1 |
| Vang_T265_cluster_1 | CP010040 | 1522890 | 1550943 | i5 |
| Vang_T265_cluster_2 | CP010041 | 609165 | 631773 | i1 |
| Vang_V01_P9A10T6_cluster_1 | NZ_MTIN01000191 | 1574 | 20776 | i1 |
| Vang_V04_P4A5T148_cluster_1 | NDHZ01000019 | 95616 | 118330 | i1 |
| Vang_V05_P4A8T149_cluster_1 | NDIA01000309 | 258 | 9569 | i1 |
| Vang_V07_P2A8T137_cluster_1 | NZ_NDIC01000339 | 1036 | 20974 | Undetermined |
| Vang_V08_P9A1T1_cluster_1 | NDID01000144 | 51 | 20548 | i1 |
| Vang_V09_P4A23P171_cluster_1 | NDIE01000046 | 1750 | 22247 | i1 |
| Vang_V10_P2A27P122_cluster_1 | NZ_NDIF01000192 | 1154 | 16735 | i1 |
| Vang_V12_P9A6T4_cluster_1 | NDIH01000087 | 1240 | 21737 | i1 |
| Vang_V14_P6S14T42_cluster_1 | NDII01000044 | 52638 | 75352 | i1 |
| Vang_V18_P1S4T112_cluster_1 | NDIL01000061 | 67478 | 90192 | i1 |
| Vang_V22_P2S10T140_cluster_1 | WXVZ01000112 | 24 | 20521 | i1 |
| Vang_VA1_cluster_1 | CP010078 | 1522758 | 1550811 | i5 |
| Vang_VA1_cluster_2 | CP010079 | 609227 | 631835 | i1 |
| Vang_VIB_18_cluster_1 | CP011436 | 1522961 | 1551014 | i5 |
| Vang_VIB_18_cluster_2 | CP011437 | 609503 | 632111 | i1 |
| Vang_VIB_93_cluster_1 | CP011438 | 1522994 | 1551047 | i5 |
| Vang_VIB_93_cluster_2 | CP011439 | 605346 | 627954 | i1 |
| Vang_VIB12_cluster_1 | CP023310 | 1355282 | 1383924 | i5 |
| Vang_VIB12_cluster_2 | CP023311 | 67291 | 90856 | Undetermined |
| Vang_VIB43_cluster_1 | CP023054 | 1860218 | 1888271 | i5 |
| Vang_VIB43_cluster_2 | CP023055 | 75342 | 97067 | i1 |
| Vch_10432-62_cluster_1 | Vch_10432-62 | 977087 | 1002578 | i1 |
| Vch_1157-74_cluster_1 | NZ_JIDL01000002 | 91544 | 117035 | i1 |
| Vch_12129-1_cluster_1 | NZ_ACFQ01000009 | 316162 | 341700 | i1 |
| Vch_1311-69_cluster_1 | NZ_JIDJ01000008 | 3627 | 26065 | i1 |
| Vch_133-73_cluster_1 | NZ_JIDK01000017 | 3583 | 26008 | i1 |
| Vch_1421-77_cluster_1 | NZ_JMBL01000002 | 93450 | 118870 | i1 |
| Vch_1421-77_cluster_2 | NZ_JMBL01000026 | 5256 | 29907 | i5 |
| Vch_1587_cluster_1 | NZ_KQ410623 | 172840 | 198403 | i1 |
| **Vch_2012EL-1759_cluster_1** | NZ_JNEW01000035 | 452809 | 478314 | i1 |
| Vch_2012Env-2_cluster_1 | NZ_JSTD01000001 | 147373 | 172866 | i1 |
| Vch_2012Env-25_cluster_1 | NZ_JSTE01000002 | 1243 | 23434 | i1 |
| Vch_2012Env-32_cluster_1 | NZ_JSTF01000001 | 1054 | 23480 | i1 |
| Vch_2012Env-9_cluster_1 | NZ_CP012997 | 12069 | 37562 | i1 |
| Vch_2012Env-92_cluster_1 | NZ_JSTJ01000001 | 52984 | 78561 | i1 |
| Vch_254-93_cluster_1 | NZ_JMBP01000002 | 80345 | 104967 | i1 |
| **Vch_2740-80_cluster_1** | NZ_KQ257330 | 194489 | 219982 | i1 |
| Vch_5473-62_cluster_1 | NZ_JIDI01000004 | 90155 | 115730 | i1 |
| Vch_571-88_cluster_1 | NZ_JIDO01000006 | 81519 | 106142 | i1 |
| Vch_623-39_cluster_1 | NZ_KQ410585 | 844624 | 870160 | i1 |
| Vch_8-76_cluster_1 | NZ_JIDN01000002 | 41708 | 66342 | i1 |
| Vch_984-81_cluster_1 | NZ_JMBM01000005 | 78833 | 104324 | i1 |
| Vch_A215_cluster_1 | NZ_CWSL01000002 | 528817 | 554218 | i1 |
| Vch_A325_cluster_1 | NZ_CWSO01000002 | 387636 | 413039 | i1 |
| Vch_AM-19226_cluster_1 | NZ_KQ257252 | 774622 | 799105 | i1 |
| Vch_BJG-01_cluster_1 | NZ_GL989136 | 4522 | 26709 | i1 |
| Vch_CP1037-10_cluster_1 | NZ_JH942316 | 147210 | 172709 | i1 |
| Vch_DL4211_cluster_1 | NZ_MOLL01000005 | 54063 | 79436 | i1 |
| Vch_DL4215_cluster_1 | NZ_MOLM01000011 | 72830 | 98377 | i1 |
| Vch_EM-1676A_cluster_1 | NZ_KB662755 | 95955 | 121508 | i1 |
| Vch_HC-1A2_cluster_1 | NZ_AJRO01000005 | 168547 | 194025 | i1 |
| Vch_HC-46B1_cluster_1 | NZ_AJSL01000011 | 666069 | 691630 | i1 |
| Vch_HE-25_cluster_1 | NZ_ALEC01000003 | 829934 | 855451 | i1 |
| Vch_HE-39_cluster_1 | NZ_AFOQ01000003 | 95553 | 121045 | i1 |
| Vch_HE-45_cluster_1 | NZ_ALED01000027 | 175381 | 200942 | i1 |
| Vch_HE-48_cluster_1 | NZ_AFOR01000005 | 570746 | 596238 | i1 |
| **Vch_I-1471_cluster_1** | NZ_CM003112 | 115142 | 140636 | i1 |
| Vch_LMA3984-4_cluster_1 | NC_017269 | 112516 | 137457 | i1 |
| **Vch_M010_cluster_1** | NZ_DS990137 | 791641 | 817134 | i1 |
| **Vch_M66-2_cluster_1** | NC_012580 | 115132 | 140625 | i1 |
| **Vch_MS6_cluster_1** | NZ_AP014525 | 108583 | 134076 | i1 |
| Vch_MZO-2_cluster_1 | NZ_KQ257215 | 786039 | 810674 | i1 |
| Vch_MZO-3_cluster_1 | NZ_KQ257285 | 227717 | 253210 | i1 |
| **Vch_N16961_cluster_1** | NC_002506 | 115141 | 140634 | i1 |
| Vch_NHCC-008D_cluster_1 | NZ_KB661860 | 149487 | 174123 | i1 |
| **Vch_O395_cluster_1** | NC_012583 | 115172 | 140648 | i1 |
| Vch_PS15_cluster_1 | NZ_AIJR01000051 | 61826 | 87223 | i1 |
| **Vch_RC27_cluster_1** | NZ_ADAI01000038 | 237344 | 254648 | i1 |
| Vch_RC385_cluster_1 | NZ_GG774556 | 161555 | 186193 | i1 |
| Vch_TM_11079-80_cluster_1 | NZ_ACHW01000028 | 13465 | 39056 | i1 |
| Vch_TMA21_cluster_1 | NZ_ACHY01000016 | 587046 | 612562 | i1 |
| Vch_V51_cluster_1 | NZ_KQ257370 | 269986 | 295482 | i1 |
| **Vch_V52_cluster_1** | NZ_KQ410497 | 202020 | 227497 | i1 |
| Vch_VC35_cluster_1 | NZ_AMBR01000011 | 93872 | 119410 | i1 |
| Vch_VL426_cluster_1 | NZ_ACHV01000002 | 317237 | 342730 | i1 |
| Vch_YB1A01_cluster_1 | NZ_LBCL01000013 | 92492 | 118084 | i1 |
| Vch_YB2A06_cluster_1 | NZ_LBFX01000005 | 200002 | 224640 | i1 |
| Vch_YB2G07_cluster_1 | NZ_LBGA01000009 | 160895 | 186448 | i1 |
| Vch_YB3B05_cluster_1 | NZ_LBGB01000001 | 93270 | 118758 | i1 |
| Vch_YB6A06_cluster_1 | NZ_LBGK01000022 | 60575 | 85196 | i1 |
| Vch_ZWU0020_cluster_1 | NZ_JRJX01000133 | 58403 | 83827 | i1 |
| Vfis_ES114_cluster_1 | NC_006840 | 1090978 | 1111242 | i1 |
| Vflu_12605_cluster_1 | NZ_CP019119 | 942357 | 967733 | i1 |
| Vflu_2013V-1049_cluster_1 | NZ_CP035778 | 712057 | 737441 | i1 |
| Vflu_2013V-1049_cluster_2 | NZ_CP035778 | 1126507 | 1150865 | i5 |
| Vflu_3663_cluster_1 | NZ_JXXQ01000023 | 29837 | 55223 | i1 |
| Vflu_539_cluster_1 | NZ_JQHX01000001 | 182202 | 207539 | i1 |
| Vflu_560_cluster_1 | NZ_JQHW01000070 | 3329 | 24018 | i1 |
| Vflu_CRA_S10_cluster_1 | NZ_QJSF01000010 | 39531 | 64915 | i1 |
| Vflu_CRA_S5_cluster_1 | NZ_QJSE01000001 | 153829 | 179271 | i1 |
| Vflu_FDAARGOS_100_cluster_1 | NZ_CP014032 | 79747 | 105066 | i1 |
| Vflu_FDAARGOS_100_cluster_2 | NZ_CP014032 | 526990 | 551357 | i5 |
| Vflu_FDAARGOS_104_cluster_1 | NZ_CP014034 | 1432865 | 1458245 | i1 |
| Vflu_I21563_cluster_1 | NZ_ASXT01000009 | 1941 | 22823 | i1 |
| Vflu_MGYG-HGUT-01703_cluster_1 | NZ_CABLBX010000005 | 201110 | 226490 | i1 |
| Vflu_NBRC_103150_cluster_1 | NZ_BCZR01000005 | 201110 | 226490 | i1 |
| Vflu_NCTC11327_cluster_1 | NZ_UHIP01000002 | 374160 | 399540 | i1 |
| Vflu_PG41_cluster_1 | NZ_ASXS01000008 | 153886 | 179278 | i1 |
| Vflu_QY27_cluster_1 | NZ_PHIF01000010 | 1801 | 22683 | i1 |
| Vflu_S1110_cluster_1 | NZ_LKHR01000074 | 1531 | 20561 | i1 |
| Vflu_S3_cluster_1 | NZ_QKNU01000001 | 200782 | 226224 | i1 |
| Vflu_ZOR0035_cluster_1 | NZ_JTLJ01000058 | 1532 | 10837 | i1 |
| Vfur_2419-04_cluster_1 | NZ_CP035694 | 149591 | 175182 | i1 |
| Vfur_2419-04_cluster_2 | NZ_CP035694 | 561177 | 585425 | i5 |
| Vfur_CIP_102972_cluster_1 | NZ_ACZP01000014 | 476112 | 501705 | i1 |
| Vfur_CIP_102972_cluster_2 | NZ_ACZP01000014 | 908790 | 933174 | i5 |
| Vfur_CRA_S16_cluster_1 | NZ_QJUD01000002 | 365063 | 389448 | i5 |
| Vfur_CRA_S16_cluster_2 | NZ_QJUD01000002 | 775668 | 801085 | i1 |
| Vfur_FDAARGOS_777_cluster_1 | NZ_CP040991 | 675610 | 701203 | i1 |
| Vfur_FDAARGOS_777_cluster_2 | NZ_CP040991 | 1108283 | 1132667 | i5 |
| Vfur_MGYG-HGUT-01713_cluster_1 | NZ_CABLCD010000014 | 476112 | 501705 | i1 |
| Vfur_MGYG-HGUT-01713_cluster_2 | NZ_CABLCD010000014 | 908790 | 933174 | i5 |
| Vfur_NCTC_11218_cluster_1 | NC_016628 | 807786 | 832165 | i5 |
| Vfur_NCTC_11218_cluster_2 | NC_016628 | 1231182 | 1256775 | i1 |
| Vfur_NCTC13120_cluster_1 | NZ_UHIT01000002 | 185717 | 211310 | i1 |
| Vfur_NCTC13120_cluster_2 | NZ_UHIT01000002 | 618389 | 642773 | i5 |
| Vfur_S0821_cluster_1 | NZ_LKHS01000001 | 166736 | 192227 | i1 |
| Vfur_S0821_cluster_2 | NZ_LKHS01000004 | 223909 | 248312 | i5 |
| Vtar_2015V-1076_cluster_1 | QKKH01000027 | 140647 | 166209 | i1 |
| Vtar_2016V-1062_cluster_1 | QKKJ01000004 | 430263 | 455825 | i1 |
| Vtar_2017V-1038_cluster_1 | QKKK01000062 | 12464 | 34653 | i1 |
| Vibrio_RC586_cluster_1 | NZ_ADBD01000008 | 490991 | 516298 | i1 |
| Vkan_10N.222.51.B7_cluster_1 | NZ_SYVH01000076 | 400 | 20815 | i1 |
| Vkan_10N.261.46.F4_cluster_1 | NZ_SYUV01000120 | 294 | 20709 | i1 |
| Vkan_10N.261.48.E7_cluster_1 | NZ_SYUU01000100 | 294 | 20709 | i1 |
| Vkan_10N.261.49.B3_cluster_1 | NZ_MCYW01000114 | 1470 | 20620 | i1 |
| Vkan_10N.286.45.A9_cluster_1 | NZ_SYUN01000121 | 1864 | 21014 | i1 |
| Vmet_06-2478_cluster_1 | NZ_LCUD01000011 | 13444 | 38719 | i1 |
| Vmet_07-2435_cluster_1 | NZ_LCUE01000001 | 458365 | 483875 | i1 |
| Vmet_08-2459_cluster_1 | NZ_CP035689 | 728 | 20242 | Undetermined |
| Vmet_2010V-1005_cluster_1 | NZ_LCUG01000003 | 288258 | 313618 | i1 |
| Vmet_2011V-1169_cluster_1 | NZ_CP035687 | 524433 | 549895 | i1 |
| Vmet_OP3H_cluster_1 | NZ_JJMN01000046 | 194695 | 219670 | i1 |
| Vmet_OYP4E03_cluster_1 | NZ_NMST01000001 | 153139 | 177720 | i1 |
| Vmet_OYP5H08_cluster_1 | NZ_NMSR01000001 | 241029 | 266564 | i1 |
| Vmet_OYP8G05_cluster_1 | NZ_NMSQ01000031 | 102495 | 124878 | i1 |
| Vmet_OYP8G09_cluster_1 | NZ_NMSP01000005 | 155536 | 181041 | i1 |
| Vmet_OYP8G12_cluster_1 | NZ_NMSO01000001 | 150088 | 175593 | i1 |
| Vmet_OYP8H05_cluster_1 | NZ_NMSN01000008 | 376406 | 401764 | i1 |
| Vmet_OYP9B03_cluster_1 | NZ_NMSM01000017 | 147302 | 172660 | i1 |
| Vmet_OYP9B09_cluster_1 | NZ_NMSL01000001 | 12593 | 34976 | i1 |
| Vmet_OYP9C12_cluster_1 | NZ_NMSK01000002 | 451593 | 477128 | i1 |
| Vmet_OYP9D09_cluster_1 | NZ_NMSJ01000004 | 163697 | 188278 | i1 |
| Vmet_OYP9E03_cluster_1 | NZ_NMSI01000003 | 105326 | 130831 | i1 |
| Vmet_OYP9E10_cluster_1 | NZ_NMSH01000001 | 238455 | 263960 | i1 |
| Vmet_RC341_cluster_1 | NZ_ACZT01000026 | 100517 | 126052 | i1 |
| Vmet_UHGG_MGYG-HGUT-02368_cluster_1 | NZ_CABMIR010000011 | 13444 | 38719 | i1 |
| Vmet_YB4D01_cluster_1 | NZ_LBGO01000023 | 309595 | 335130 | i1 |
| Vmet_YB5B04_cluster_1 | NZ_LBGP01000005 | 148515 | 174065 | i1 |
| Vmet_YB5B06_cluster_1 | NZ_LBGQ01000013 | 155536 | 181041 | i1 |
| Vmet_YB9D03_cluster_1 | NZ_LBGR01000015 | 319910 | 345415 | i1 |
| Vmim_2011V-1073_cluster_1 | NZ_CP035683 | 809397 | 834963 | i1 |
| Vmim_523-80_cluster_1 | NZ_JIDP01000001 | 433740 | 459226 | i1 |
| Vmim_ATCC_33654_cluster_1 | NZ_VWYC01000053 | 3870 | 26269 | i1 |
| Vmim_CAIM_1882_cluster_1 | NZ_AWWY01000162 | 39661 | 61836 | i1 |
| Vmim_CAIM_1883_cluster_1 | NZ_AWWL01000160 | 39661 | 61839 | i1 |
| Vmim_CAIM_602_cluster_1 | NZ_AOMO01000027 | 111805 | 137369 | i1 |
| Vmim_FDAARGOS_112_cluster_1 | NZ_CP014042 | 475100 | 500652 | i1 |
| Vmim_FDAARGOS_113_cluster_1 | NZ_LOSJ02000001 | 1228242 | 1253728 | i1 |
| Vmim_MB-451_cluster_1 | NZ_ADAF01000002 | 149796 | 175182 | i1 |
| Vmim_N2733_cluster_1 | NZ_VSGA01000014 | 353916 | 379282 | i1 |
| Vmim_N2763_cluster_1 | NZ_VSGV01000016 | 60225 | 85786 | i1 |
| Vmim_N2781_cluster_1 | NZ_VSHK01000012 | 154643 | 180175 | i1 |
| Vmim_N2789_cluster_1 | NZ_VSHS01000014 | 399902 | 425465 | i1 |
| Vmim_N2790_cluster_1 | NZ_VSHT01000021 | 56899 | 82451 | i1 |
| Vmim_N2810_cluster_1 | NZ_VSIF01000059 | 58376 | 83932 | i1 |
| Vmim_N2816_cluster_1 | NZ_VSIL01000006 | 231 | 25697 | i1 |
| Vmim_NCTC11435_cluster_1 | NZ_UHIG01000002 | 163762 | 189325 | i1 |
| Vmim_SCCF01_cluster_1 | NZ_CP016383 | 2715760 | 2745562 | i5 |
| Vmim_SCCF01_cluster_2 | NZ_CP016384 | 659939 | 685502 | i1 |
| Vmim_SX-4_cluster_1 | NZ_GL989609 | 156411 | 181897 | i1 |
| Vmim_VM223_cluster_1 | NZ_ADAJ01000007 | 823638 | 849014 | i1 |
| Vmim_VM573_cluster_1 | NZ_ACYV01000016 | 243704 | 269192 | i1 |
| Vmim_VM603_cluster_1 | NZ_ACYU01000018 | 146104 | 171627 | i1 |
| Vord_Q67_cluster_1 | CP022742 | 82842 | 106527 | i1 |
| Vpch_07-2425_cluster_1 | QKKO01000039 | 541 | 25960 | i1 |
| Vpch_2014V-1107_cluster_1 | NZ_QKKP01000020 | 29943 | 55437 | i1 |
| Vpch_2016V-1091_cluster_1 | NZ_QKKQ01000017 | 339067 | 364574 | i1 |
| Vpch_2016V-1114_cluster_1 | NZ_QKKS01000038 | 35605 | 61189 | i1 |
| Vpch_2017V-1144_cluster_1 | QKKV01000065 | 34426 | 59831 | i1 |
| Vpch_2017V-1176_cluster_1 | QKKW01000042 | 45711 | 71195 | i1 |
| Vpch_490-93_cluster_1 | NZ_JIDQ01000039 | 15218 | 28865 | i1 |
| Vpch_87395_cluster_1 | NZ_KB661497 | 137852 | 163271 | i1 |
| Vpch_877-163_cluster_1 | NZ_LBNV01000038 | 690 | 26183 | i1 |
| Vpch_HE-09_cluster_1 | NZ_GL988768 | 137933 | 163430 | i1 |
| Vpch_HE-16_cluster_1 | NZ_JH941441 | 170631 | 196050 | i1 |
| Vpch_VCC19_cluster_1 | NZ_ATEV02000022 | 44942 | 70436 | i1 |
| Vphl_19-021-D1_cluster_1 | NZ_CP046411 | 596838 | 631721 | i1 |
| Vphl_19-021-D1_cluster_2 | NZ_CP046412 | 110726 | 136760 | i5 |
| Vphl_2012AW-0154_cluster_1 | NZ_CP035701 | 1557429 | 1588237 | i1 |
| Vphl_2012AW-0154_cluster_2 | NZ_CP035702 | 245337 | 271371 | i5 |
| Vphl_FDAARGOS_115_cluster_1 | NZ_CP014046 | 2608313 | 2643182 | i1 |
| Vphl_FDAARGOS_115_cluster_2 | NZ_CP014047 | 335104 | 361138 | i5 |
| Vphl_RIMD_2210633_cluster_1 | NC_004603 | 1492843 | 1524323 | i1 |
| Vphl_RIMD_2210633_cluster_2 | NC_004605 | 1079346 | 1105380 | i5 |
| Vsco_FP3289_cluster_1 | NZ_MDCJ01000007 | 664946 | 684117 | i1 |
| Vsco_LMG_19158_cluster_1 | NZ_AFWE01000218 | 47698 | 66869 | i1 |
| Vsco_VS-05_cluster_1 | NZ_CP016416 | 75035 | 103452 | i1 |
| Vvul_93U204_cluster_1 | NZ_CP009262 | 1061342 | 1088516 | i5 |
| Vvul_ATCC_27562_cluster_1 | NZ_CP012882 | 1162924 | 1188990 | i5 |
| Vvul_CECT_4999_cluster_1 | NZ_CP014637 | 1000161 | 1025944 | i5 |
| Vvul_CG100_cluster_1 | PDGD01000023 | 4957 | 33028 | i5 |
| Vvul_CG100_cluster_2 | PDGD01000078 | 170041 | 196100 | i5 |
| Vvul_FDAARGOS_116_cluster_1 | NZ_LOSI02000001 | 1573946 | 1600012 | i5 |
| Vvul_FORC_054_cluster_1 | NZ_CP019121 | 358848 | 388660 | i5 |
| Vvul_FORC_054_cluster_2 | NZ_CP019122 | 1066263 | 1093420 | i5 |
| Vvul_LSU2098_cluster_1 | PDFY01000204 | 121300 | 147366 | i5 |
| Vvul_NCTC11066_cluster_1 | NZ_UHIK01000002 | 426702 | 452768 | i5 |
| Vvul_YJ016_cluster_1 | NC_005140 | 1086439 | 1112504 | i5 |

***** *V. cholerae* strains in the pandemic clade are shown in bold.
