# Supplementary material for: Pandemic Vibrio cholerae acquired competitive traits from an environmental Vibrio species
Source: Life Sci Alliance. 2022 Nov 29;6(2):e202201437. doi: 10.26508/lsa.202201437 (PMC9711863; doi:10.26508/lsa.202201437)
Supplement: Supplementary file 2 [file LSA-2022-01437_TableS2.docx]

**Supplemental Table S2. Auxiliary T6SS gene clusters identified in *V. anguillarum* strains.**

| **Strain/cluster** | **contig** | **start** | **end** |
| --- | --- | --- | --- |
| Vang 030305-1/5 Accessory Aux1 | RCUB01000087 | 3527 | 26 |
| Vang 030305-1/5 Accessory Aux1 2 | RCUB01000113 | 34745 | 30294 |
| Vang 030305-1/5 Aux1 | RCUB01000044 | 1 | 2666 |
| Vang 030305-1/5 Aux2 | RCUB01000032 | 19 | 6076 |
| Vang 030305-1/5 Aux4 | RCUB01000031 | 4485 | 1 |
| Vang 030305-1/5 Aux4 2 | RCUB01000171 | 1 | 2517 |
| Vang 12B09 Accessory Aux1 | AJYV02000004 | 1030748 | 1034237 |
| Vang 12B09 Aux1 | AJYV02000004 | 643072 | 638574 |
| Vang 12B09 Aux2 | AJYV02000053 | 120354 | 128435 |
| Vang 178/90 Accessory Aux1 | CP011471 | 882098 | 886324 |
| Vang 178/90 Aux1 | CP011471 | 467475 | 463512 |
| Vang 178/90 Aux2 | CP011470 | 2846809 | 2838719 |
| Vang 261/91 Accessory Aux1 | CP010033 | 884603 | 888829 |
| Vang 261/91 Aux1 | CP010033 | 469984 | 466021 |
| Vang 261/91 Aux2 | CP010032 | 2846660 | 2838570 |
| Vang 425 Accessory Aux1 | CP020533 | 83182 | 87408 |
| Vang 425 Aux1 | CP020533 | 716863 | 712900 |
| Vang 425 Aux2 | CP020534 | 2748390 | 2740300 |
| Vang 4299 Accessory Aux1 | CP011459 | 885662 | 889918 |
| Vang 4299 Aux1 | CP011459 | 469183 | 467340 |
| Vang 4299 Aux2 | CP011458 | 2855270 | 2786460 |
| Vang 4299 Aux2 2 | CP011458 | 3223696 | 3228053 |
| Vang 4299 Aux4 | CP011458 | 3355036 | 3360654 |
| Vang 51/82/2 Accessory Aux1 | CP010043 | 882034 | 886260 |
| Vang 51/82/2 Aux1 | CP010043 | 467410 | 463447 |
| Vang 51/82/2 Aux2 | CP010042 | 2846792 | 2838702 |
| Vang 531Ac Accessory Aux1 | VSLE01000024 | 23469 | 19243 |
| Vang 531Ac Aux1 | VSLE01000060 | 8821 | 7039 |
| Vang 531Ac Aux2 | VSLE01000035 | 8895 | 805 |
| Vang 601/90 Accessory Aux1 | CP010077 | 882048 | 886274 |
| Vang 601/90 Aux1 | CP010077 | 467425 | 463679 |
| Vang 601/90 Aux2 | CP010076 | 2846691 | 2838601 |
| Vang 6018/1 Accessory Aux1 | CP010292 | 881692 | 885918 |
| Vang 6018/1 Aux1 | CP010292 | 467071 | 463108 |
| Vang 6018/1 Aux2 extraction | CP010291 | 2846791 | 2838701 |
| Vang 775 Accessory Aux1 | CP002285 | 886601 | 890827 |
| Vang 775 Aux1 | CP002285 | 471946 | 467983 |
| Vang 775 Aux2 | CP002284 | 2848565 | 2840475 |
| Vang 87-9-116 Accessory Aux1 | CP021981 | 340682 | 344908 |
| Vang 87-9-116 Aux1 | CP021981 | 1143127 | 1139164 |
| Vang 87-9-116 Aux2 | CP021980 | 443326 | 451416 |
| Vang 87-9-117 Accessory Aux1 | CP010047 | 879655 | 883881 |
| Vang 87-9-117 Aux1 | CP010047 | 465027 | 461064 |
| Vang 87-9-117 Aux2 | CP010046 | 2846819 | 2838729 |
| Vang 90-11-286 Accessory Aux1 | CP011461 | 1043968 | 1048281 |
| Vang 90-11-286 Aux1 | CP011461 | 645505 | 640177 |
| Vang 90-11-286 Aux2 | CP011460 | 435360 | 443936 |
| Vang 90-11-286 Aux4 | CP011461 | 481611 | 475279 |
| Vang 90-11-287 Accessory Aux1 | CP011476 | 882011 | 886237 |
| Vang 90-11-287 Aux1 | CP011476 | 467392 | 463429 |
| Vang 90-11-287 Aux2 | CP011475 | 2846826 | 2838736 |
| Vang 9014/8 Accessory Aux1 | CP010039 | 880881 | 885107 |
| Vang 9014/8 Aux1 | CP010039 | 466262 | 462299 |
| Vang 9014/8 Aux2 | CP010038 | 2846779 | 2838689 |
| Vang 91-7-154 Accessory Aux1 | CP010083 | 882172 | 886398 |
| Vang 91-7-154 Aux1 | CP010083 | 467549 | 463586 |
| Vang 91-7-154 Aux2 | CP010082 | 2847878 | 2839788 |
| Vang 91-8-178 Accessory Aux1 | CP010035 | 884589 | 888815 |
| Vang 91-8-178 Aux1 | CP010035 | 469969 | 466006 |
| Vang 91-8-178 Aux2 | CP010034 | 2846690 | 2838600 |
| Vang 96F Accessory Aux1 | AEZA01000028 | 48735 | 44533 |
| Vang 96F Aux1 | AEZA01000061 | 1 | 1938 |
| Vang 96F Aux2 | AEZA01000002 | 3967 | 12048 |
| Vang A023 Accessory Aux1 | CP010037 | 884662 | 886496 |
| Vang A023 Aux1 | CP010037 | 469277 | 466057 |
| Vang A023 Aux2 | CP010036 | 2846912 | 2838822 |
| Vang ATCC 14181 Accessory Aux1 | MCJC01000146 | 3469 | 717 |
| Vang ATCC 14181 Accessory Aux1 2 | MCJC01000047 | 30258 | 34623 |
| Vang ATCC 14181 Aux2 | MCJC01000111 | 25907 | 16288 |
| Vang ATCC_14181 Aux1 | MCJC01000134 | 1 | 2665 |
| Vang ATCC_14181 Aux4 | MCJC01000033 | 4395 | 1 |
| Vang ATCC_14181 Aux4 2 | MCJC01000148 | 2549 | 597 |
| Vang ATCC-68554 Accessory Aux1 | CP023209 | 644902 | 640676 |
| Vang ATCC-68554 Aux1 | CP023209 | 63020 | 66983 |
| Vang ATCC-68554 Aux2 | CP023208 | 2621717 | 2613627 |
| Vang Ba35 Accessory Aux1 | CP010031 | 884602 | 888828 |
| Vang Ba35 Aux1 | CP010031 | 469996 | 466033 |
| Vang Ba35 Aux2 | CP010030 | 2846866 | 2838776 |
| Vang CNEVA NB11008 Accessory Aux1 | CP022104 | 346917 | 350358 |
| Vang CNEVA NB11008 Aux1 | CP022104 | 1062399 | 1058276 |
| Vang CNEVA NB11008 Aux2 | CP022103 | 515092 | 523552 |
| Vang CNEVA NB11008 Aux4 | CP022104 | 307035 | 313374 |
| Vang CNEVA NB11008 Aux4 2 | CP022103 | 3023224 | 3016884 |
| Vang DSM 21597 Accessory Aux1 | CP010085 | 909880 | 913474 |
| Vang DSM 21597 Aux1 | CP010085 | 503800 | 500786 |
| Vang DSM 21597 Aux2 | CP010084 | 2738031 | 2729947 |
| Vang DSM 21597 Aux3 | CP010084 | 3303415 | 3304992 |
| Vang FF-167 Accessory Aux1 | NZ_AJYR02000016 | 10023 | 14257 |
| Vang FF-167 Aux1 | NZ_AJYR02000096 | 12892 | 40793 |
| Vang FF-167 Aux2 | NZ_AJYR02000081 | 2618 | 10702 |
| Vang FF-93 Accessory Aux1 | AJYT02000218 | 26163 | 22674 |
| Vang FF-93 Aux1 | AJYT02000071 | 1 | 1842 |
| Vang FF-93 Aux2 | AJYT02000196 | 39718 | 31636 |
| Vang FS-144 Accessory Aux1 | AJYU02000067 | 18966 | 15477 |
| Vang FS-144 Aux1 | AJYU02000055 | 6429 | 8311 |
| Vang FS-144 Aux2 | AJYU02000274 | 17251 | 9424 |
| Vang FS-238 Accessory Aux1 | NZ_AJYS02000215 | 98422 | 102034 |
| Vang FS-238 Aux1 | NZ_AJYS02000153 | 1 | 6323 |
| Vang FS-238 Aux2 | NZ_AJYS02000047 | 7723 | 16290 |
| Vang HI610 Accessory Aux1 | CP011463 | 921372 | 924782 |
| Vang HI610 Accessory Aux1 2 | CP011462 | 3244961 | 3240732 |
| Vang HI610 Aux1 | CP011463 | 513298 | 509450 |
| Vang HI610 Aux2 | CP011462 | 2788113 | 2778494 |
| Vang HI610 Aux4 | CP011462 | 3226829 | 3231266 |
| Vang HI618 Accessory Aux1 | MNLD01000073 | 11596 | 16034 |
| Vang HI618 Accessory Aux1 2 | MNLD01000098 | 102 | 799 |
| Vang HI618 Aux1 | MNLD01000193 | 1 | 1418 |
| Vang HI618 Aux2 | MNLD01000040 | 19 | 2811 |
| Vang HI618 Aux2 2 | MNLD01000114 | 4692 | 20 |
| Vang HI618 Aux2 3 | MNLD01000116 | 1 | 4324 |
| Vang HI618 Aux4 | MNLD01000047 | 24 | 5642 |
| Vang J360 Accessory Aux1 | CP034673 | 511578 | 520500 |
| Vang J360 Aux1 | CP034673 | 104925 | 97099 |
| Vang J360 Aux2 | CP034672 | 2087702 | 2097321 |
| Vang J360 Aux4 | CP034673 | 1090473 | 1084140 |
| Vang J360 Aux4 2 | CP034672 | 602723 | 593822 |
| Vang JLL237 Accessory Aux1 | NZ_CP022102 | 329533 | 335082 |
| Vang JLL237 Aux1 | NZ_CP022102 | 1102640 | 1098517 |
| Vang JLL237 Aux2 | NZ_CP022101 | 470910 | 478996 |
| Vang JLL237 Aux4 | NZ_CP022102 | 941050 | 934718 |
| Vang LMG12010 Accessory Aux1 | CP011469 | 879487 | 883713 |
| Vang LMG12010 Aux1 | CP011469 | 464856 | 460893 |
| Vang LMG12010 Aux2 | CP011468 | 2846867 | 2838777 |
| Vang M3 Accessory Aux1 | CP006700 | 886600 | 890826 |
| Vang M3 Aux1 | CP006700 | 471921 | 467958 |
| Vang M3 Aux2 | CP006699 | 2848354 | 2840264 |
| Vang M93 Accessory Aux1 | NOWD01000047 | 14046 | 16798 |
| Vang M93 Accessory Aux1 2 | NOWD01000003 | 41362 | 37034 |
| Vang M93 Aux1 | NOWD01000034 | 9077 | 1251 |
| Vang M93 Aux2 | NOWD01000046 | 24040 | 33659 |
| Vang M93 Aux4 | NOWD01000056 | 2573 | 7184 |
| Vang M93 Aux4 2 | NOWD01000044 | 26986 | 23792 |
| Vang MHK3 Accessory Aux1 | CP022469 | 1083853 | 1079624 |
| Vang MHK3 Aux1 | CP022469 | 397640 | 401826 |
| Vang MHK3 Aux2 | CP022468 | 681593 | 690144 |
| Vang NB10 Accessory Aux1 | LK021129 | 353827 | 358029 |
| Vang NB10 Aux1 | LK021129 | 1122810 | 1118847 |
| Vang NB10 Aux2 | LK021130 | 437549 | 445639 |
| Vang NCTC12159 Accessory Aux1 | UGPJ01000002 | 478764 | 482334 |
| Vang NCTC12159 Aux1 | UGPJ01000002 | 127208 | 118020 |
| Vang NCTC12159 Aux2 | UGPJ01000001 | 1758159 | 1766243 |
| Vang NCTC12159 Aux3 | UGPJ01000001 | 233769 | 232192 |
| Vang PF4 Accessory Aux1 | CP023290 | 530236 | 525968 |
| Vang PF4 Aux1 | CP023290 | 455405 | 451859 |
| Vang PF4 Aux2 | CP023291 | 2511803 | 2503239 |
| Vang PF430-3 Accessory Aux1 | CP011467 | 912165 | 916433 |
| Vang PF430-3 Aux1 | CP011467 | 508911 | 504747 |
| Vang PF430-3 Aux2 | CP011466 | 2787018 | 2778454 |
| Vang PF7 Accessory Aux1 | CP011465 | 902865 | 906354 |
| Vang PF7 Aux1 | CP011465 | 416902 | 421617 |
| Vang PF7 Aux2 | CP011464 | 2736260 | 2727799 |
| Vang RV22 Accessory Aux1 | AEZB01000258 | 3681 | 6433 |
| Vang RV22 Accessory Aux1 2 | AEZB01000003 | 37500 | 41828 |
| Vang RV22 Aux1 | AEZB01000008 | 1 | 2191 |
| Vang RV22 Aux2 | AEZB01000033 | 70518 | 60896 |
| Vang RV22 Aux4 | AEZB01000031 | 4429 | 2430 |
| Vang S2 2/9 Accessory Aux1 | CP011473 | 864232 | 867829 |
| Vang S2 2/9 Aux1 | CP011473 | 460456 | 456313 |
| Vang S2 2/9 Aux2 | CP011472 | 2586534 | 2578448 |
| Vang S3 4/9 Accessory Aux1 | CP022100 | 346666 | 350979 |
| Vang S3 4/9 Aux1 | CP022100 | 1166033 | 1161910 |
| Vang S3 4/9 Aux2 | CP022099 | 422697 | 430783 |
| Vang T265 Accessory Aux1 | CP010041 | 884698 | 888924 |
| Vang T265 Aux1 | CP010041 | 470081 | 466118 |
| Vang T265 Aux2 | CP010040 | 2846797 | 2838707 |
| Vang VA1 Accessory Aux1 | CP010079 | 884760 | 888986 |
| Vang VA1 Aux1 | CP010079 | 470140 | 466177 |
| Vang VA1 Aux2 | CP010078 | 2846642 | 2838552 |
| Vang VIB 18 Accessory Aux1 | CP011437 | 885039 | 889265 |
| Vang VIB 18 Aux1 | CP011437 | 470419 | 466456 |
| Vang VIB 18 Aux2 | CP011436 | 2846730 | 2838640 |
| Vang VIB 93 Accessory Aux1 | CP011439 | 880881 | 885107 |
| Vang VIB 93 Aux1 | CP011439 | 466262 | 462299 |
| Vang VIB 93 Aux2 | CP011438 | 2846779 | 2838689 |
| Vang VIB12 Accessory Aux1 | CP023311 | 475613 | 481398 |
| Vang VIB12 Aux1 | CP023311 | 1211411 | 1204783 |
| Vang VIB12 Aux4 | CP023311 | 467815 | 474147 |
| Vang VIB43 Accessory Aux1 | CP023055 | 346429 | 355351 |
| Vang VIB43 Aux1 | CP023055 | 1090147 | 1082321 |
| Vang VIB43 Aux2 | CP023054 | 503316 | 512934 |
| Vang VIB43 Aux4 | CP023055 | 920288 | 913955 |
| Vang VIB43 Aux4 2 | CP023054 | 2266238 | 2258603 |
| Vang_V01_P9A10T6 Accessory Aux1 | NZ_MTIN01000101 | 15192 | 18690 |
| Vang_V01_P9A10T6 Aux1 | NZ_MTIN01000074 | 1 | 6341 |
| Vang_V01_P9A10T6 Aux2 | NZ_MTIN01000163 | 1 | 8073 |
| Vang_V01_P9A10T6 Aux4 | NZ_MTIN01000034 | 34108 | 40438 |
| Vang_V02_P2A34T13 Accessory Aux1 | NZ_PXJL01000105 | 1 | 2669 |
| Vang_V02_P2A34T13 Aux1 | NZ_PXJL01000160 | 1 | 24291 |
| Vang_V02_P2A34T13 Aux2 | NZ_PXJL01000270 | 5352 | 13673 |
| Vang_V02_P2A34T13 Aux4 | NZ_PXJL01000222 | 33976 | 40309 |
| Vang_V04_P4A5T148 Accessory Aux1 | NDHZ01000081 | 23590 | 28488 |
| Vang_V04_P4A5T148 Aux1 | NDHZ01000019 | 91180 | 118330 |
| Vang_V04_P4A5T148 Aux2 | NDHZ01000013 | 31979 | 40318 |
| Vang_V05_P4A8T149 Accessory Aux1 | NDIA01000313 | 1910 | 4325 |
| Vang_V05_P4A8T149 Aux1 | NDIA01000157 | 1 | 13896 |
| Vang_V05_P4A8T149 Aux2 | NDIA01000030 | 25127 | 33466 |
| Vang_V07_P2A8T137 Accessory Aux1 | NZ_NDIC01000182 | 1 | 1646 |
| Vang_V07_P2A8T137 Accessory Aux1 2 | NZ_NDIC01000352 | 1222 | 3065 |
| Vang_V07_P2A8T137 Aux1 | NZ_NDIC01000267 | 1 | 4514 |
| Vang_V07_P2A8T137 Aux2 | NZ_NDIC01000317 | 184 | 7978 |
| Vang_V07_P2A8T137 Aux4 | NZ_NDIC01000140 | 33982 | 40315 |
| Vang_V08_P9A1T1 Accessory Aux1 | NDID01000056 | 1 | 3244 |
| Vang_V08_P9A1T1 Aux1 | NDID01000001 | 7095 | 11607 |
| Vang_V08_P9A1T1 Aux2 | NDID01000041 | 20431 | 29002 |
| Vang_V09_P4A23P171 Accessory Aux1 | NDIE01000002 | 252881 | 256394 |
| Vang_V09_P4A23P171 Aux1 | NDIE01000002 | 1 | 4337 |
| Vang_V09_P4A23P171 Aux2 | NDIE01000045 | 25106 | 33189 |
| Vang_V10_P2A27P122 Accessory Aux1 | NZ_NDIF01000204 | 729 | 4194 |
| Vang_V10_P2A27P122 Aux1 | NZ_NDIF01000032 | 1 | 7573 |
| Vang_V10_P2A27P122 Aux2 | NZ_NDIF01000046 | 5196 | 13517 |
| Vang_V10_P2A27P122 Aux4 | NZ_NDIF01000297 | 9649 | 15982 |
| Vang_V12_P9A6T4 Accessory Aux1 | NDIH01000007 | 851 | 4370 |
| Vang_V12_P9A6T4 Aux1 | NDIH01000054 | 167687 | 173584 |
| Vang_V12_P9A6T4 Aux2 | NDIH01000027 | 260 | 8816 |
| Vang_V14_P6S14T42 Accessory Aux1 | NDII01000095 | 25332 | 29013 |
| Vang_V14_P6S14T42 Aux1 | NDII01000044 | 48202 | 75352 |
| Vang_V14_P6S14T42 Aux2 | NDII01000108 | 31979 | 40318 |
| Vang_V18_P1S4T112 Accessory Aux1 | NDIL01000125 | 1 | 2923 |
| Vang_V18_P1S4T112 Aux1 | NDIL01000061 | 63042 | 90192 |
| Vang_V18_P1S4T112 Aux2 | NDIL01000114 | 23046 | 31385 |
| Vang_V22_P2S10T140 Accessory Aux1 | WXVZ01000163 | 709 | 4174 |
| Vang_V22_P2S10T140 Aux1 | WXVZ01000045 | 1 | 6403 |
| Vang_V22_P2S10T140 Aux2 | WXVZ01000278 | 104 | 8425 |
| Vang_V22_P2S10T140 Aux4 | WXVZ01000172 | 34101 | 40434 |
