# Supplementary material for: Pandemic Vibrio cholerae acquired competitive traits from an environmental Vibrio species
Source: Life Sci Alliance. 2022 Nov 29;6(2):e202201437. doi: 10.26508/lsa.202201437 (PMC9711863; doi:10.26508/lsa.202201437)
Supplement: Supplementary file 4 [file LSA-2022-01437_TableS3.docx]

**Supplemental Table S3. Representative *V. cholerae* effector types**

| **T6SS Cluster** | **Effector Type** | **Strain** | **Common Name** | **Locus Tag** | **RefSeq Accession** |
| --- | --- | --- | --- | --- | --- |
| Large | A | N16961 | *vgrG-3* | VCA0123 | WP_000113295.1 |
| Large | B | 8-76 |  | DN40_RS15540/ DN40_RS15545 | WP_032474496.1/  WP_000264537.1 |
| Large | C | 623-39 |  | A59_RS11670 | WP_000113306.1 |
| Large | D | HE-45 |  | VCHE45_RS04895 | WP_000113307.1 |
| Large | E | DL4211 |  | BLX44_RS02325 | WP_080497324.1 |
| Large | F | TMA21 |  | VCB_RS11800 | WP_000113301.1 |
| Large | G | 133-73 |  | DN37_RS05965 | WP_000113299.1 |
| Large | I | 877-163 |  | F546_RS08515 | WP_158003294.1 |
| Large | J | 1421-77 |  | DN41_RS15390 | None associated* |
| Large | K | A325 |  | ALE21_RS05965 | None associated* |
| Large | L | 490-93 |  | DA89_RS06285 | WP_000113297.1 |
| Large | M | OYP2A12 |  | CGT70_07560 | WP_240308516.1 |
| Aux1 | A | N16961 | *tseL* | VC1418 | WP_000376836.1 |
| Aux1 | C | 8-76 |  | DN40_RS13315 | WP_032474653.1 |
| Aux2 | A | N16961 | *vasX* | VCA0020 | WP_000070352.1 |
| Aux2 | B | TMA21 |  | VCB_RS11320 | WP_000097988.1 |
| Aux2 | C | 984-81 |  | DN42_RS11035 | WP_000104678.1 |
| Aux2 | D | 133-73 |  | DN37_RS01310 | WP_032471792.1 |
| Aux2 | E | DL4211 |  | BLX44_RS07700 | WP_076025196.1 |
| Aux3 | N/A | N16961 | *tseH* | VCA0285 | WP_000132861.1 |
| Aux4 | N/A | 984-81 | *tpeV* | DN42_RS00580 | WP_032481643.1 |

***** No RefSeq Accession number associated with any of the effectors of the given type in any *V. cholerae* strain. Amino acid sequence obtained by translating the nucleotide sequence.
