# Supplementary material for: Pandemic Vibrio cholerae acquired competitive traits from an environmental Vibrio species
Source: Life Sci Alliance. 2022 Nov 29;6(2):e202201437. doi: 10.26508/lsa.202201437 (PMC9711863; doi:10.26508/lsa.202201437)
Supplement: Supplementary file 6 [file LSA-2022-01437_TableS5.docx]

**Supplemental Table S5. *Vibrio* genomes used for the multi-species tree (Fig. 3, 6A)**

| **Sequence Name** | **RefSeq or GenBank Accession** |
| --- | --- |
| Vibrio anguillarum strain JLL237 | GCF_002211985.1 |
| Vibrio anguillarum strain S2 2/9 | GCA_001989895.1 |
| Vibrio anguillarum strain VIB43 | GCF_002287545.1 |
| Vibrio cholerae strain 12129(1) | GCF_000174115.1 |
| Vibrio cholerae strain 2012Env-9 | GCF_000788715.2 |
| **Vibrio cholerae strain N16961** | GCF_000006745.1 |
| **Vibrio cholerae strain O395** | GCF_000021625.1 |
| Vibrio fluvialis strain 12605 | GCF_001952955.1 |
| Vibrio fluvialis strain FDAARGOS_100 | GCF_002953375.1 |
| Vibrio furnissii strain 2419-04 | GCF_009665335.1 |
| Vibrio furnissii strain FDAARGOS_777 | GCF_006364355.1 |
| Vibrio kanaloae strain 5S-149 | GCF_000272165.2 |
| Vibrio kanaloae strain 10N.222.51.B7 | GCF_005146725.1 |
| Vibrio metoecus strain 08-2459 | GCF_009665275.1 |
| Vibrio metoecus strain 2011V-1169 | GCF_009665255.1 |
| Vibrio mimicus strain 2011V-1073 | GCF_009665195.1 |
| Vibrio mimicus strain FDAARGOS_112 | GCF_001558475.2 |
| Vibrio mimicus strain SCCF01 | GCF_001767355.1 |
| Vibrio ordalii strain Q67 | GCA_002257545.1 |
| Vibrio paracholerae strain 2014V-1107 | GCF_003311945.1 |
| Vibrio paracholerae strain 2017V-1176 | GCF_003312095.1 |
| Vibrio parahaemolyticus strain 19-021-D1 | GCF_009734325.1 |
| Vibrio parahaemolyticus strain 2012AW-0154 | GCF_009665495.1 |
| Vibrio scophthalmi strain VS-05 | GCF_001687805.1 |
| Vibrio scophthalmi strain VS-12 | GCF_001685465.1 |
| Vibrio tarriae 2016V-1062 | GCF_003311825.1 |
| Vibrio tarriae 2017V-1038 | GCF_003311805.1 |
| Vibrio vulnificus strain 93U204 | GCF_000746665.1 |
| Vibrio vulnificus strain CECT 4999 | GCF_002215135.1 |
| Vibrio vulnificus strain FORC_054 | GCF_002863725.1 |

***** *V. cholerae* strains in the pandemic clade are shown in bold.
