# Supplementary material for: Pandemic Vibrio cholerae acquired competitive traits from an environmental Vibrio species
Source: Life Sci Alliance. 2022 Nov 29;6(2):e202201437. doi: 10.26508/lsa.202201437 (PMC9711863; doi:10.26508/lsa.202201437)
Supplement: Supplementary file 7 [file LSA-2022-01437_TableS6.docx]

**Supplemental Table S6. Bacterial strains and plasmids.**

| **Strain or Plasmid** | **Genotype/Description** | **Internal**  **Strain**  **Ref.** | **Reference and/or Source** |
| --- | --- | --- | --- |
| *V. cholerae* V52 | O37 serogroup strain isolated in Sudan in 1968 from a clinical sample; Sm^R^ | DU167 | John Mekalanos (Harvard Medical School, Boston, MA, USA) |
| *V. cholerae* V52 Δ*vasK* | V52 deleted for the T6SS membrane complex component *vasK*; Sm^R^ | DU168 | (MacIntyre et al. 2010) |
| *V. cholerae* DL4211 | O123 serogroup strain, isolated from the Rio Grande river delta (USA) in 2008; Aux3-naïve; Sm^R^ | DU608 | Daniele Provanzano (UTRGV, Brownsville, TX, USA |
| *V. cholerae* DL4211 Δ*vasK* | DL4211 deleted for the T6SS membrane complex component *vasK*; Sm^R^ | DU357 | (Unterweger et al. 2012) |
| *V. anguillarum* VIB43 | *Vibrio anguillarum* O1 serogroup strain isolated from a diseased *Dicentrarchus labrax* (European bass); Rif^R^ | FJS300 | Hans Rediers (KU-Leuven, Leuven, Belgium) |
| *E. coli* DH5α λpir | F^-^ endA1 glnV44 thi-1 recA1 relA1 gyrA96 deoR nupG φ80lacZΔM15 Δ(lacZYA-argF) U169 hsdR17 (r_K_^-^ m_K_^+^) phoA, λ^-^ | FJS010 | (Platt et al. 2000) |
| *E. coli* BL21(DE3) pLysS | Str. B F^–^ *ompT* *gal* *dcm* *lon* *hsdS_B_*(*r_B_*^–^*m_B_*^–^) λ(DE3 [*lacI* *lacUV5*-*T7p07* *ind1* *sam7* *nin5*]) [*malB*^+^]_K-12_(λ^S^) pLysS[*T7p20* *ori*_p15A_];Cm^R^ | HM016 | (Studier and Moffatt 1986) |
| *E. coli* BL21(DE3) pLysS ; pET26b(+) ; pET22b(+) | BL21(DE3) carrying empty pET26b(+) and empty pET22b(+) expression vectors; Cm^R^, Kan^R^, Amp^R^ | HM020 | This study |
| *E. coli* BL21(DE3) pLysS ; pET26b(+)-*tseL*-6xHis ; pET22b(+) | BL21(DE3) strain for the expression of TseL-6xHis ; Cm^R^, Kan^R^, Amp^R^ | FJS444 | This study |
| *E. coli* BL21(DE3) pLysS ; pET26b(+) ; pET22b(+)-*tsiV1*-6xHis | BL21(DE3) strain for the expression of TsiV1-6xHis ; Cm^R^, Kan^R^, Amp^R^ | FJS445 | This study |
| *E. coli* BL21(DE3) pLysS ; pET26b(+)-Aeff^V09^-6xHis ; pET22b(+) | BL21(DE3) strain for the expression of Aeff^V09^-6xHis ; Cm^R^, Kan^R^, Amp^R^ | FJS446 | This study |
| *E. coli* BL21(DE3) pLysS ; pET26b(+) ; pET22b(+)-Aimm^V09^-6xHis | BL21(DE3) strain for the expression of Aimm^V09^-6xHis ; Cm^R^, Kan^R^, Amp^R^ | FJS447 | This study |
| *E. coli* BL21(DE3) pLysS ; pET26b(+)-*tseL*-6xHis ; pET22b(+)-*tsiV1-*6xHis | BL21(DE3) strain for the co-expression of TseL-6xHis and TsiV1-6xHis ; Cm^R^, Kan^R^, Amp^R^ | FJS448 | This study |
| *E. coli* BL21(DE3) pLysS ; pET26b(+)-*tseL*-6xHis ; pET22b(+)- Aimm^V09^*-*6xHis | BL21(DE3) strain for the co-expression of TseL-6xHis and Aimm^V09^-6xHis ; Cm^R^, Kan^R^, Amp^R^ | FJS449 | This study |
| *E. coli* BL21(DE3) pLysS ; pET26b(+)-Aeff^V09^-6xHis ; pET22b(+)- Aimm^V09^-6xHis | BL21(DE3) strain for the co-expression of Aeff^V09^-6xHis and Aimm^V09^-6xHis ; Cm^R^, Kan^R^, Amp^R^ | FJS450 | This study |
| *E. coli* BL21(DE3) pLysS ; pET26b(+)-Aeff^V09^-6xHis ; pET22b(+)- *tsiV1-*6xHis | BL21(DE3) strain for the co-expression of Aeff^V09^-6xHis and TsiV1-6xHis ; Cm^R^, Kan^R^, Amp^R^ | FJS451 | This study |
| Plasmids | | | |
| pET26b(+)-*tseL*-6xHis | pET26b(+) with inserted copy of *tseL* | FJS439 | This study |
| pET22b(+)-*tsiV1*-6xHis | pET22b(+) with inserted copy of *tsiV1* | FJS442 | This study |
| pET26b(+)-Aeff^V09^-6xHis | pET26b(+) with inserted copy of Aeff^V09^ | FJS438 | This study |
| pET22b(+)-Aimm^V09^-6xHis | pET22b(+) with inserted copy of Aimm^V09^ | FJS441 | This study |
