# Supplementary material for: Pandemic Vibrio cholerae acquired competitive traits from an environmental Vibrio species
Source: Life Sci Alliance. 2022 Nov 29;6(2):e202201437. doi: 10.26508/lsa.202201437 (PMC9711863; doi:10.26508/lsa.202201437)
Supplement: Supplementary file 8 [file LSA-2022-01437_TableS7.docx]

**Supplemental Table S7. Primers.**

| **Ref** | **Name** | **Sequence*** |
| --- | --- | --- |
| FJS328 | pET26_N16961_*tseL_*His_F | ggccatggatatcggaattaattcgATGGATTCATTTAATTATTGCGTG |
| FJS329 | pET26_N16961_*tseL_*His_R | gatctcagtggtggtggtggtggtg***tcctcctcc***TCTTATTTGCACCTTGATTTCATC |
| FJS326 | pET26_V09_Aeff*_*His_F | ggccatggatatcggaattaattcgATGGATTCATTTAACCATTGCG |
| FJS327 | pET26_V09_Aeff*_*His_R | gatctcagtggtggtggtggtggtg***tcctcctcc***TTGTAGTTGTTCCTTAATTTCATCAG |
| FJS330 | pET22_N16961_*tsiV1_*His_F | ggccatggatatcggaattaattcgATGAAGTTATTGAATAATCTTGCAATAAAAAAG |
| FJS331 | pET22_N16961_*tsiV1_*His_R | gatctcagtggtggtggtggtggtg***tcctcctcc***ATTATCATCAGATACCACTGCTG |
| FJS332 | pET22_V09_Aimm*_*His_F | ggccatggatatcggaattaattcgATGAAGTTATTGAATAACCTCGC |
| FJS333 | pET22_V09_Aimm*_*His_R | gatctcagtggtggtggtggtggtg***tcctcctcc***TTGCGCTACAGCGACTTG |
| FJS334 | pET22_pET26_insert_verification_F | TGTGAGCGGATAACAATTCCC |
| FJS335 | pET22_pET26_insert_verification_R | AGCCAACTCAGCTTCCTTTC |

* For all Gibson assembly primers, overlap regions are shown in lowercase, and added glycine linker is shown in ***bold/italic***.
